# Comparative metabolism of cellulose, sophorose and glucose in *Trichoderma reesei* using high-throughput genomic and proteomic analyses

**DOI:** 10.1186/1754-6834-7-41

**Published:** 2014-03-21

**Authors:** Lilian dos Santos Castro, Wellington Ramos Pedersoli, Amanda Cristina Campos Antoniêto, Andrei Stecca Steindorff, Rafael Silva-Rocha, Nilce M Martinez-Rossi, Antonio Rossi, Neil Andrew Brown, Gustavo H Goldman, Vitor M Faça, Gabriela F Persinoti, Roberto Nascimento Silva

**Affiliations:** 1 Department of Biochemistry and Immunology, Ribeirão Preto Medical School, University of São Paulo, 14049-900 Ribeirão Preto, SP, Brazil; 2 Departamento de Biologia Celular, Universidade de Brasília, Asa Norte, 70910-900 Brasília, DF, Brazil; 3 Department of Genetics, Ribeirão Preto Medical School, University of São Paulo, 14049-900 Ribeirão Preto, SP, Brazil; 4 Faculdade de Ciências Farmacêuticas de Ribeirão Preto, Universidade de São Paulo, São Paulo, and Laboratório Nacional de Ciência e Tecnologia do Bioetanol, Campinas, Brazil

**Keywords:** Trichoderma reesei, RNA-seq, DIGE, Cellulases, Bioethanol

## Abstract

**Background:**

The filamentous fungus *Trichoderma reesei* is a major producer of lignocellulolytic enzymes utilized by bioethanol industries. However, to achieve low cost second generation bioethanol production on an industrial scale an efficient mix of hydrolytic enzymes is required for the deconstruction of plant biomass. In this study, we investigated the molecular basis for lignocellulose-degrading enzyme production *T. reesei* during growth in cellulose, sophorose, and glucose.

**Results:**

We examined and compared the transcriptome and differential secretome (2D-DIGE) of *T. reesei* grown in cellulose, sophorose, or glucose as the sole carbon sources. By applying a stringent cut-off threshold 2,060 genes were identified as being differentially expressed in at least one of the respective carbon source comparisons. Hierarchical clustering of the differentially expressed genes identified three possible regulons, representing 123 genes controlled by cellulose, 154 genes controlled by sophorose and 402 genes controlled by glucose. Gene regulatory network analyses of the 692 genes differentially expressed between cellulose and sophorose, identified only 75 and 107 genes as being specific to growth in sophorose and cellulose, respectively. 2D-DIGE analyses identified 30 proteins exclusive to sophorose and 37 exclusive to cellulose. A correlation of 70.17% was obtained between transcription and secreted protein profiles.

**Conclusions:**

Our data revealed new players in cellulose degradation such as accessory proteins with non-catalytic functions secreted in different carbon sources, transporters, transcription factors, and CAZymes, that specifically respond in response to either cellulose or sophorose.

## Background

The growing worldwide demand for energy and the desire to reduce dependency on finite fossil fuels has increased interest in alternative energy sources, especially liquid biofuels such as bioethanol and biodiesel. Ethanol obtained from lignocellulosic, non-food, feedstocks (for example, sugarcane bagasse or wheat straw) represents an attractive alternative due to its applicability in existing motor vehicles. In addition, the combustion of lignocellulosic-derived ethanol is considered cleaner than oil-based fuels [1]. Lignocellulosic biomass locks away approximately half of the energy produced by plants during photosynthesis and is the most abundant renewable organic carbon resource on Earth. Lignocellulose predominately consists of three polymers that are tightly interlinked, cellulose, hemicellulose and lignin, which correspond to approximately 98% of lignocellulose dry weight [2].

The production of fuel ethanol from lignocellulose requires biomass pretreatment, cellulose hydrolysis, hexose fermentation, separation, effluent treatment, and depending on the raw material, additional costs may occur [3]. In recent years, new technologies have been developed for the pretreatment of sugarcane bagasse such as the application of novel enzymes to increase the saccharification of cellulose/hemicellulose and specialized fermentation technologies, aiding in the development of second-generation (2G) bioethanol [4].

The filamentous fungus *Trichoderma reesei* is one of the main producers of cellulases and hemicellulases used in industrial scale [5] and is especially important for the production of 2G biofuels from lignocellulose [6]. Despite *T. reesei* being the most prominent lignocellulosic degrader among the genus *Trichoderma*, this species has a reduced number of cellulolytic enzymes compared to other lignocellulosic fungi [7]. This ability is attributed to *T. reesei* possessing efficient systems for the transport of nutrients and the induction/secretion of cellulases. Subsequently, the study of the cellulolytic system in *T. reesei* is of substantial interest to industrial biotechnology.

The *T. reesei* cellulolytic system consists of at least three different types of enzymes: exoglucanases (cellobiohydrolases EC 3.2.1.91), endoglucanases (EC 3.2.1.4) and β-glucosidases (EC 3.2.1.21), which occur in various isoforms [8]. *T. reesei* produces at least two exo-acting cellobiohydrolases (CEL7A and CEL6A), five endo-acting cellulases (CEL7B, CEL5A, CEL5B, CEL12A, CEL45A), two characterized β-glucosidases (CEL3A and CEL1A), and an additional five predicted β-glucosidases (CEL3B, CEL3D, CEL1B, CEL3C, CEL3E) [9]. Besides the classic cellulases, new players involved in cellulose degradation were recently described in *T. reesei*, such as the expansin-like proteins swollenin (SWOI) and expansin/family 45 endoglucanase-like (EEL1, EEL2, and EEL3) [7,10]. In addition, GH61 polysaccharide monooxygenases (PMOs), which were recently re-annotated as AA9 (Auxiliary family activity 9, http://www.cazy.org), have been shown to enhance lignocellulose degradation by an oxidative mechanism [9].

The production of the main cellulases by *T. reesei* is controlled by a sophisticated regulation system that avoids energy expenditure on unrequired processes when readily metabolisable carbon sources are present [6]. Since the 1960s when Mary Mandels and Elwyn T. Reese [11], raised the question ‘Cellulases are adaptive enzymes, but the natural substrate – cellulose – is insoluble. So how does induction occur?’ many studies have been conducted in an attempt to discover the natural inducer of cellulase formation [12-14]. It is now known that expression of cellulolytic genes in *T. reesei* are induced in the presence of cellulose and several disaccharides such as cellobiose (β-D-glucopyranosyl-(1 → 4)-β-D-glucopyranose), δ-cellobiono-1,5-lactone (β-D-glucopyranosyl-(1 → 4)-D-glucono-1,5-lactone), lactose (β-D-galactopyranosyl-(1 → 4)-D-glucose) and sophorose (2-O-β-D-glucopyranosyl-α-D-glucose)[15]. Sophorose is the strongest cellulase inducer and is considered to be a possible natural inducer. It is assumed that sophorose is formed by *T. reesei* during cellulose hydrolysis by a transglycosylation reaction [16]. However, additional low-molecular weight compounds have been reported to promote cellulase gene expression, such as l-arabitol and l-sorbose [17]. In contrast, the presence of easily metabolisable carbon sources such as glucose and fructose, represses the expression of cellulolytic genes [18].

The regulation of cellulase gene expression occurs at the transcriptional level in a coordinated manner and is dependent on the presence of the inducer [19]. This regulation is driven by specific transcriptional factors (TFs) that bind to cellulase gene promoters acting either in a positive or a negative way. So far, at least three transcriptional activators XYR1, ACE2, the HAP2/3/5 complex, as well as the two repressors CRE1 and ACE1 are involved in the regulation of cellulase gene expression in *T. reesei*[20].

Despite extensive studies attempting to answer the question raised by Mandels and Reese, neither the nature of the inducer nor how *T. reesei* senses the inducer and relays the cellulase induction signal, have been elucidated. In this study we report a comparison of the transcriptome (RNAseq) and secretome (two-dimensional Fluorescence Difference Gel Electrophoresis (2-D DIGE)) of *T. reesei* grown on cellulose, sophorose and glucose, in attempt to understand the molecular basis of lignocellulose-degrading enzyme induction. Our results provide new insights and revealed new players in cellulose degradation such as proteins with non-catalytic functions secreted in different carbon sources, transporters, transcription factors, carbohydrate active enzyme (CAZymes), and the regulatory network of *T. reesei* in response to cellulose and sophorose. These data will contribute to the development of industrial *T. reesei* strains by engineering its metabolism to produce high levels of cellulases for plant cell-wall degradation.

## Results

### Global gene expression profiles of *Trichoderma reesei* grown in three different carbon sources

*T. reesei* QM9414 was grown directly in three different carbon sources; glucose, sophorose, and cellulose as described (see Methods). We previously demonstrated the growth profiles and glucose consumption of *T. reesei* QM9414 in the presence of cellulose and glucose [21]. Based on these data, we designed the strategy to pool the time points of each condition before the sequencing. Nine barcoded libraries were sequenced using the Illumina Hiseq 2000 System, generating approximately 117 million 100-bp paired-end reads corresponding to 23.32 GB of sequence data (Additional file 1: Table S2). Reads were mapped to the *T. reesei* QM6a reference genome available from JGI (*Trichoderma reesei* v2.0) using the Bowtie aligner. Overall, 68% of reads mapped to the reference genome (Additional file 1: Table S2). There was a high correlation (Pearson correlation, *r*^2^ ≥ 0.71) between the three biological replicates of each condition used in the transcriptional analysis (Additional file 2: Figure S1A-B). After sample normalization, boxplots were constructed in order to determine if the conditions are comparable and the results are shown (see Additional file 2, Figure S1 C-D). The boxplots showed that both normalized samples and raw data displayed the same plot profile and no significant statistical difference (*P* <0.05), demonstrating that the samples are comparable.

**Figure 1:**
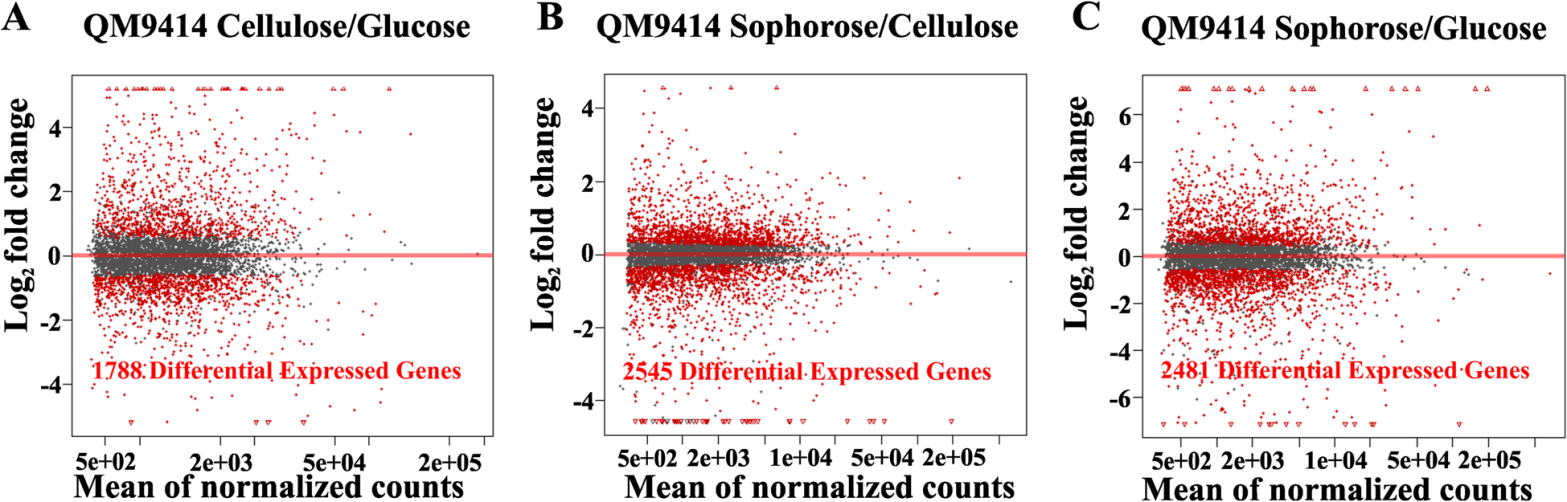
**Comparison of full-genome expression profiles of QM9414 strain grown in cellulose, sophorose, and glucose as the carbon source, measured by RNA-seq. (A)** Cellulose/glucose. **(B)** Cellulose/sophorose. **(C)** Sophorose/glucose. Differentially expressed genes identified by DESeq package are plotted in red (*P* <0.05).

The *T. reesei* gene expression profiles obtained from the different carbon sources were analyzed using R Bioconductor DESeq. Of the 9,129 genes encoded by the *T. reesei* genome, 1,788 genes were identified as being differentially expressed (*P* <0.05) on glucose/cellulose, 2,545 genes on sophorose/cellulose and 2,481 genes on sophorose/glucose (Figure 1A-C).

**Figure 2:**
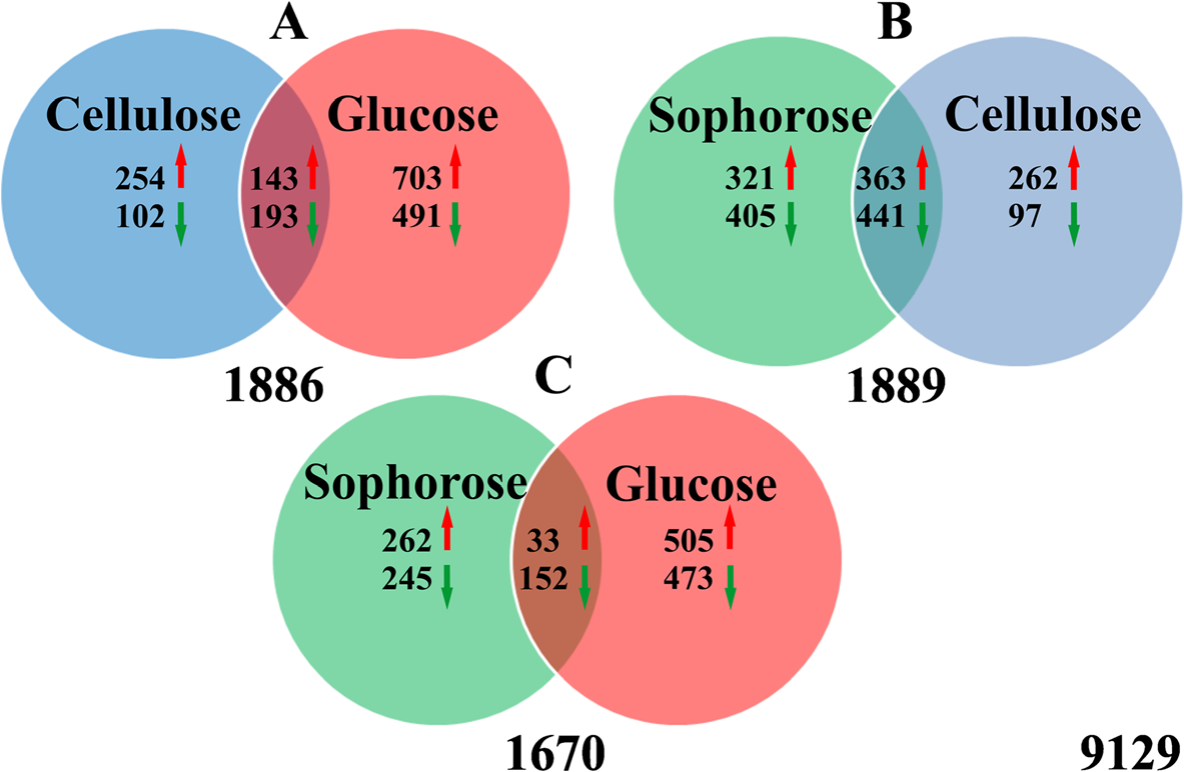
**Venn diagram representing the number of differentially expressed genes in the QM9414 strain. (A)** QM9414 strain growth in cellulose and glucose as carbon source. **(B)** QM9414 strain growth in cellulose and sophorose. **(C)** QM9414 strain growth in sophorose and glucose. The red arrows indicate the number of upregulated genes and green arrows the number of downregulated genes in the conditions analyzed. The numbers below the given Venn diagram represent the total number of regulated genes. The number in the lower right of rectangle indicates the number of transcripts in the *T. reesei* genome. Thresholds for calling differentially expressed genes were (*P* ≤0.05) and ≥ two-fold change, that is, log_2_-fold change ≥1 or ≤ -1.

Appling a two-fold change (that is, log_2_ fold change ≥1 or ≤ -1) and an adjusted *P*-value ≤0.05 as a threshold, 2,060 genes were identified as differentially expressed in at least one of the respective carbon source comparisons. Figure 2A shows that 1,886 genes were differentially expressed in glucose and in cellulose, as represented by 703 and 491 genes being up- and downregulated exclusively in glucose, and 254 and 102 genes being up- and downregulated exclusively in cellulose, respectively. On the other hand, 1,889 genes were differentially expressed in sophorose and in cellulose, with 321 and 405 being up- or downregulated in sophorose, and 262 and 97 being up- or downregulated in cellulose, respectively (Figure 2B). Yet, in glucose and sophorose 1,670 genes were modulated, with 262 and 245 being up- or downregulated in sophorose, and 505 and 473 up- or downregulated genes in glucose, respectively (Figure 2C). Interestingly, the number of transcriptionally modulated genes in sophorose (726) was greater than that in cellulose (359), when both were compared to glucose.

**Figure 3:**
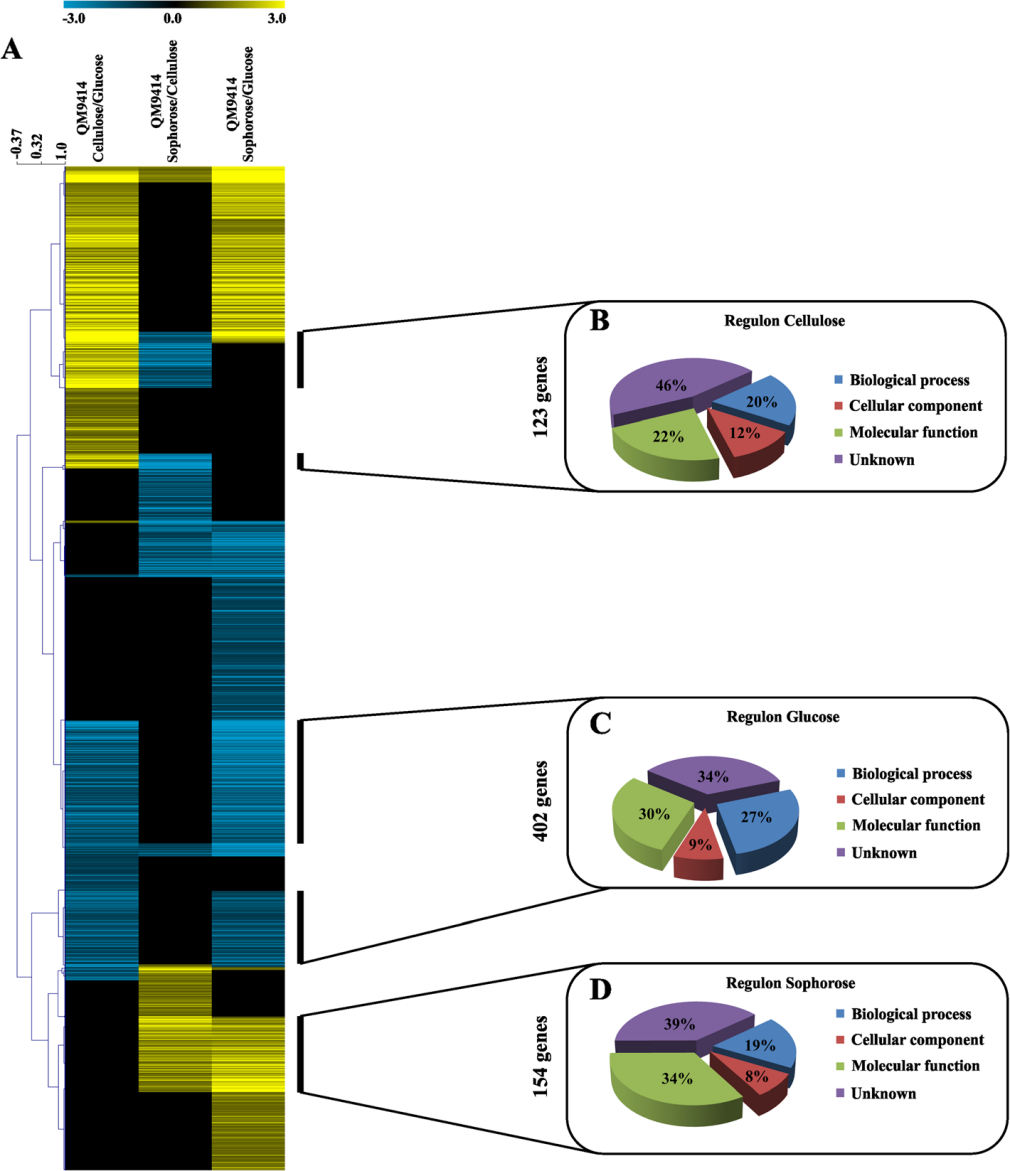
**Gene expression profile of *T. reesei*, QM9414 strain, during grown in the presence of cellulose, sophorose and glucose as the carbon source.** Expression scale is represented as Log_2_ Fold Change. **(A)** Hierarchical clustering analysis was performed using Mev v.4.6.1, with the average linkage method for cluster generation, and uncentered correlation as the similarity metric. Euclidian distance was used to measure the differences in gene expression among the 2,060 genes and the groups (QM9414 Cellulose/Glucose; QM9414 Sophorose/Cellulose and QM9414 Sophorose/Glucose) based on the distance between the centroids of the groups, *P* <0.05. **(B)** Genes upregulated by cellulose, **(C)** genes upregulated by glucose and **(D)** genes upregulated by sophorose. In **(B)**, **(C)** and **(D)** the summary of the Gene Ontology annotation are also represented.

Hierarchical clustering of the 2,060 differentially expressed genes identified in the comparisons cellulose versus glucose (cel/glu), sophorose versus cellulose (soph/cel) and sophorose versus glucose (soph/glu), allowed the identification of three possible regulons, representing 123 genes modulated by cellulose, 154 genes modulated by sophorose and 402 genes modulated by glucose, totaling 679 genes (Figure 3A; Additional file 3: Table S3). Gene Ontology (GO) annotation of the 679 carbon source-specific genes revealed that 46%, 34% and 39% of the genes from the cellulose, glucose and sophorose regulons respectively were genes of unknown function. These results emphasize the potential for the discovery of genes involved in the cellulase production in *T. reesei* during growth under inducing or repressing conditions.

In order to further evaluate the carbon source-specific regulons shown in Figure 3, the top 10 genes differentially expressed on cellulose, glucose, and sophorose were identified (Table 1). The top 10 upregulated genes in cellulose included the glycoside hydrolases (GH) GH5, GH31 and GH16, the carbohydrate esterase CE5, an oxidase, a specific Major facilitator superfamily (MFS) permease and five proteins of unknown function (Table 1). The top 10 upregulated genes in sophorose included a GH76 hydrolase, four oxidoreductases, two MFS permeases and three proteins of unknown function (Table 1). It is interesting to note that there are more GHs in the top 10 upregulated genes of cellulose than in sophorose. As expected, the top 10 differentially expressed genes in glucose did not show any genes encoding hydrolytic enzymes (Table 1). These results indicate a specific gene expression in response to the available carbon source in *T. reesei*.

**Table 1:** **Log**_**2 **_**fold change (FC) of the top 10 genes differentially expressed in cellulose, glucose and sophorose**

**Condition**	**Protein ID**	**Name**	**GO term**	**FC Cel/Glu**	**FC Sph/Cel**	**FC Sph/Glu**
**Cellulose**	69957	MFS permease	Cellular component	11.001	-3.673	0
	56996	GH5 β-Mannanase MAN1	Biological process	10.849	-5.403	0
	69944	GH31 α-xylosidase/α-glucosidase	Biological process	10.500	-2.007	0
	73632	CE5 acetyl xylan esterase AXE1	Molecular function	8.060	-1.025	0
	108642	Unknown protein	Unknown	3.644	-8.621	0
	112258	Unknown protein	Unknown	4.665	-8.069	0
	124079	Multicopper oxidases	Molecular function	3.787	-7.762	0
	123236	SSCRP	Unknown	2.666	-7.713	0
	119552	Unique protein	Unknown	3.884	-7.090	0
	55886	GH16 glucan endo-1,3(4)-β-D-glucosidase	Unknown	4.086	-7.067	0
**Glucose**	79816	Unknown protein; secreted	Molecular function	-5.793	0	-9.354
	70520	short chain dehydrogenase/reductase	Molecular function	-4.333	0	-9.263
	23382	Aldehyde reductase AKR7	Molecular function	-4.028	0	-9.213
	30759	Zinc-containing alcohol dehydrogenase superfamily	Molecular function	-3.684	0	-8.508
	123084	Chloroperoxidase	Molecular function	-4.587	0	-8.369
	122998	Unknown protein	Unknown	-3.640	0	-7.637
	69115	Dienelactone hydrolase	Unknown	-4.827	0	-7.496
	81525	Isoflavone reductase	Molecular function	-4.430	0	-7.448
	81586	Unknown protein	Unknown	-2.903	0	-7.338
	76641	MFS permease	Cellular component	-4.454	0	-7.309
**Sophorose**	106164	short chain dehydrogenase/reductase	Molecular function	0	2.026	10.007
	59628	Unknown protein	Unknown	0	3.846	5.711
	48444	MFS maltose permease	Cellular component	0	2.109	5.524
	5345	FAD-containing oxidoreductase	Molecular function	0	2.579	5.443
	122087	Unknown protein	Cellular component	0	2.898	5.225
	21876	Zinc-binding oxidoreductase	Molecular function	0	5.034	3.828
	22915	Glucose oxidase	Molecular function	0	1.156	4.544
	110267	Unknown protein	Biological process	0	4.486	1.287
	60945	MFS permease	Cellular component	0	1.341	4.451
	55802	GH76 α-1,6-mannanase	Molecular function	0	1.956	4.441

GO, Gene Ontology; MFS, Major facilitator superfamily; GH, glycoside hyrolase; CE, carbohydrate esterase; AXE, acetyl xylan esterase; SSCRP, Small secreted cysteine-rich protein; Cel, cellulose; Glu, glucose; Sph, sophorose.

### CAZYome

**Figure 4:**
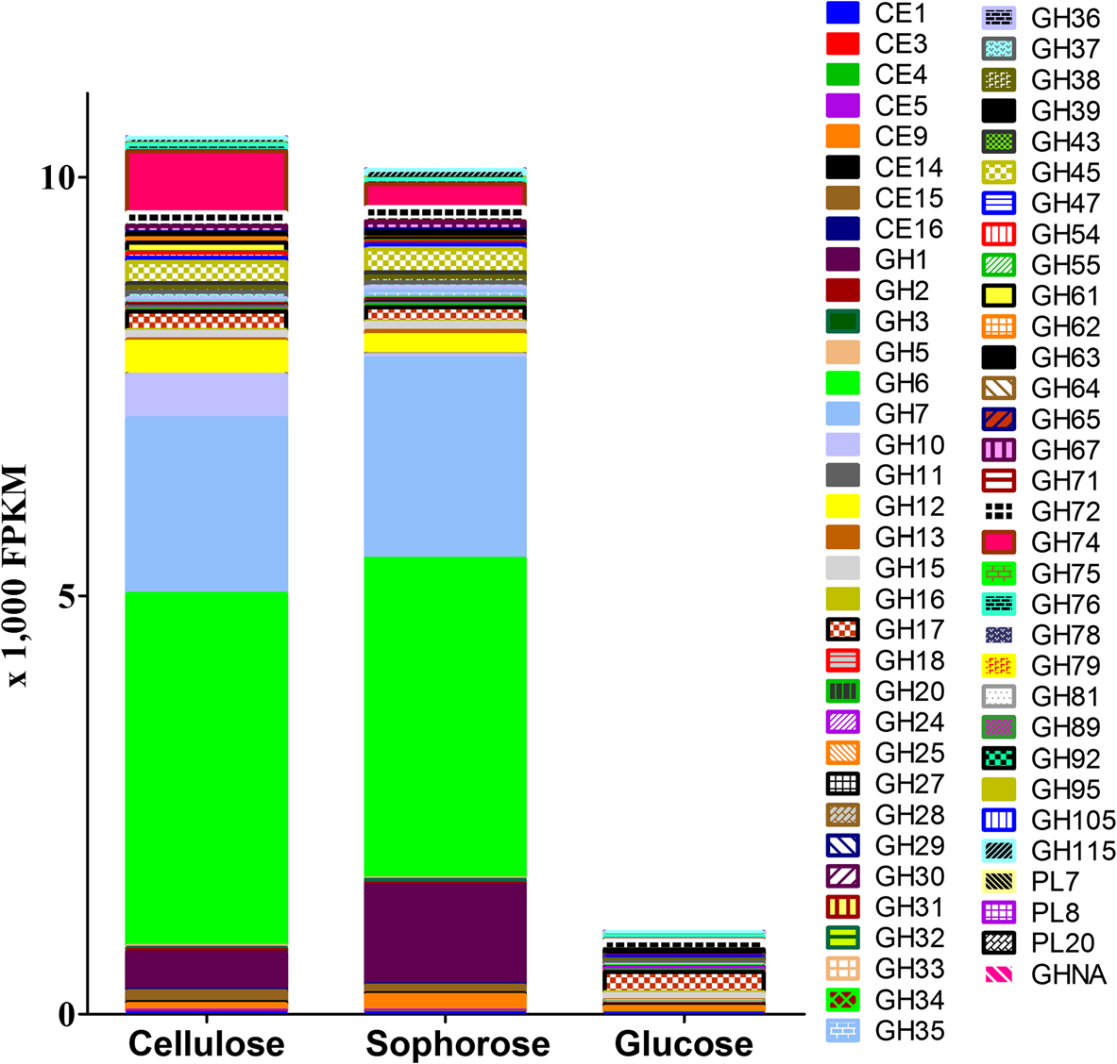
**Carbohydrate active enZymes (CAZy) genes and expression data from RNA-seq analysis.** Fragments per kilobase of exon per million fragments mapped **(**FPKM) means for each glycolic hydrase (GH) family when cultured in glucose, cellulose and sophorose. CAZy classification was performed based on re-annotation of CAZy genes of *T. reesei* according to Hakkinen *et al*. [9].

The mean FPKM (fragments per kilobase of exon per million fragments mapped) for all the genes within a single GH family were calculated. The total of all the FPKM means for each GH family when cultured in glucose, cellulose and sophorose were utilized to demonstrate the overall enzymatic potential and global transcriptional response (Figure 4). During growth in glucose the overall transcription of GH encoding genes was low, whereas growth in the presence of cellulose or sophorose resulted in a dramatic induction of a wide array of GH families, reflecting the transcriptional induction of the CAZYome. Similarly, cellulose and sophorose resulted in a greater transcriptional induction of cellobiohydrolase members from the GH6 and GH7 families.

Looking in more detail, Table S4 (Additional file 4) shows the enzymes that are differentially upregulated in sophorose and cellulose. Twenty GHs and one CE were upregulated in response to the presence of sophorose whereas 23 GHs and two CEs were induced in the presence of cellulose (Additional file 4: Table S4A and B). Interestingly, genes of enzymes involved in xylan degradation, such as xylanases *(xyn2*, *xyn3*, *xyn4*), acetyl xylan esterase (*axe1*), xyloglucanase (*cel74a*), α-xylosidase (GH31) and arabinoxylans degradation, such as arabinofuranosidase (ABF1 and ABF2) were preferentially expressed in the presence of cellulose but not in sophorose (Additional file 4: Table S4B). Furthermore, a polysaccharide monooxygenase (*cel61A*) was upregulated only in cellulose, in accordance with a role in the cellulose oxidation process. These results were also observed by Bischof *et al*. [22] when transcriptional data from wheat straw was compared to lactose. On the other hand, eight genes encoding α - and β-glucosidases (including *cel3c*, *cel3b* and *cel1b*), and a candidate for α-amylase and α-1,6-mannanase (while on cellulose a β-mannanase was expressed), were upregulated in sophorose (Additional file 4: Table S4A). Interestingly, in both sophorose and cellulose, enzymes that degrade trehalose were induced indicating that the fungus may catabolize stored trehalose, producing glucose, during cellulase production.

When comparing the fold change in gene expression among the three conditions we observed that even in the presence of glucose, 17 GHs were upregulated (Additional file 4: Table S4C). These genes encoded for enzymes such as endoglucanase (*cel5b*), β-1,4-glucanase (GH5), β-1,3-glucanosyltransferase (GH72), and an uncharacterized GH (Trire2_121136) appeared not to be subject to carbon catabolite repression.

### Transcription factors

Table S5 (see Additional file 5) shows the TF encoding genes that were induced in each condition. In this analysis, 7 TF encoding genes were upregulated in cellulose, 18 in sophorose and 18 other TF genes were specific to glucose. Within this group, we focused on the TF genes that were upregulated depending on the carbon source. For example, Trire2_105269 showed a high level of expression in the presence of cellulose, whereas Trire2_123881 showed a high level of expression in sophorose, and Trire2_ID 112499 was upregulated by glucose (Additional file 5: Table S5). TF encoding genes from the Zn2Cys6 subgroup known as C2H2 (one of the most common type of transcription factors found in eukaryotes) were only induced in the presence of cellulose and sophorose, but not in glucose-grown cells (Additional file 5: Table S5), suggesting a specific response of C2H2 to the presence of cellulase inducer molecules. TF encoding genes from the bZIP family, on the other hand, showed higher expression in cellulose (Trire2_110152), but were also present in sophorose (Trire2_73654) and glucose (Trire2_119759) (Additional file 5: Table S5).

The expression of TF encoding genes already characterized as being involved in the regulation of the expression of cellulases and hemicellulases is shown in Table 2. Among the positively acting TFs (XYR1, ACE2, CLR-1, CLR-2, and BglR), the gene for XYR1 showed the highest expression level, followed by CLR-1 and BglR (Table 2). The TF genes ACE2 and CLR-2 showed no significant modulation in expression (*P* <0.05) between the various carbon sources. These results reinforce the hypothesis that XYR1 is the major positive regulator of cellulases and hemicellulase gene expression. On the other hand, the TFs that negatively regulate hydrolytic enzyme gene transcription, such as ACE1 and CREI, showed a lower level of expression, compared to the positive-acting TFs, or were not transcriptionally modulated depending upon the carbon source, suggesting that these TFs may act in a cooperative manner or have a more effective mode of action. In addition, the gene for PacC (pH-responsive transcription factor) was regulated in a carbon source manner, showing a higher expression level in the presence of cellulose (Table 2). Other TFs that have been shown to have a regulatory role, such as HAP2/3 and AreA, showed no significant modulation in gene expression in any condition (log_2_ > 1 and *P* <0.05). Taken together, our results depict a complex system of TFs that regulate the expression of hydrolytic enzymes, while also revealing additional, uncharacterized, TFs that appear to play a role.

**Table 2:** **Log**_**2 **_**fold change of characterized transcriptional factor genes involved in the regulation of cellulase and hemicellulase genes**

**Protein ID**	**Name**	**Cel/Glu**	**Soph/Cel**	**Soph/Glu**
122208	XYR1	6.062	2.087	8.131
78445	ACEII	NS	0.069	NS
27600	CLR-1	2.263	0.516	2.766
26163	CLR2	NS	NS	NS
52368	BglR	1.492	NS	1.570
120117	ACEI	0.807	-0.674	NS
120117	CREI	0.807	-0.674	NS
124286	HAP2	NS	0.455	NS
121080	HAP3	NS	NS	NS
76817	AreA	NS	0.458	NS
120698	PacC	2.123	-0.538	NS

NS, non-significant at *P* <0.05; Cel, cellulose; Glu, glucose; Soph, sophorose

### Transporters

Genes that encode proteins involved in transport comprise about 5% (459 genes) of the *T. reesei* genome. Our results show that among these genes, 14 were regulated exclusively by cellulose, 14 by sophorose, and 30 by glucose, applying an adjusted *P*-value <0.05 as thresholds (Additional file 6: Table S6).

The MFS (Major facilitator superfamily) permeases are the most abundant proteins in the three analyzed conditions. These proteins enable the transport of essential nutrients and ions, plus the excretion of end products of metabolism and cell-environment communication [23]. The gene encoding for the MFS permease (Trire2_69957) that was specifically highly upregulated in cellulose may be involved in the transport of disaccharides, due to a high similarity with a putative maltose permease of the human pathogenic fungus *Talaromyces marneffei*[24]. Another maltose permease encoding gene (Trire2_48444) was also highly induced by sophorose. Conversely, the MFS permease gene Trire2_76641 was expressed at a higher level in glucose than on sophorose or cellulose (Table S6). A BlastP analysis of this MFS permease showed 85% sequence identity to a synaptic vesicle transporter SVOP and also shared structural similarity to the human glucose transporter 1 (Glut1) [25]. Interestingly, a gene encoding a potential galactose permease (Trire2_62380) that was specifically expressed in glucose and a MFS permease encoding gene (Trire2_76800) that was induced by cellulose, both resembled the 19 *Saccharomyces cerevisiae* transporters that when deleted, contribute to the total loss of hexose uptake [26].

In order to identify the MFS permeases shared by cellulose and sophorose, the expression results were normalized with the glucose condition (Table 3). From 85 MFS permeases annotated in the *T. reesei* genome, 22 of them seem to be shared by cellulose and sophorose (Table 3). Among them, the most expressed were: *crt1*, which has been shown to be required by *T. reesei* for growth in cellulose and lactose, but not in xylan [27]; *hxt1*, a glucose permease; the MFS gene Trire2_50894, a high affinity glucose transporter [28]; and an MFS gene related to cellulose signaling (Trire2_79202) [29]. Interestingly, the recently described *stp1*, which is involved in cellobiose and glucose transport [27], showed a higher level of expression in sophorose than in cellulose (Table 3), indicating a complex regulation on cellobiose/sophorose uptake by *T. reesei*.

**Table 3:** **The log**_**2 **_**fold change of sharing MFS permeases genes in cellulose and sophorose conditions**

**Protein ID**	**Description**	**Cellulose**	**Sophorose**
3405	MFS permease (Crt1)	7.802	9.893
22912	MFS permease (glucose permease HXT1)	7.142	6.368
50894	MFS permease	6.683	7.069
79202	MFS permease, associated with cellulose signalling	5.742	7.646
44175	MFS H + sugar transporter	4.413	4.102
121482	MFS permease	4.364	3.887
67752	MFS permease	4.085	3.919
68812	MFS permease	3.921	3.002
104549	MFS permease	3.871	4.482
47710	MFS permease (Stp1)	3.629	5.065
78833	MFS permease (fucose permease)	3.340	4.967
62502	MFS permease	2.971	3.137
54632	MFS permease	2.841	2.940
80026	MFS permease	2.718	3.073
50618	MFS permease	2.439	2.932
119789	MFS permease	2.135	2.055
106330	MFS permease	1.875	3.465
67334	MFS permease	1.740	2.234
61350	MFS permease	1.665	2.995
65153	MFS permease	1.663	2.731
68813	MFS permease	1.392	1.102
64874	MFS toxin efflux pump	1.155	1.017

Another family of proteins that showed carbon source-dependent transcriptional regulation were the ABC (ATP binding cassette) transporters, which were highly upregulated in cellulose and sophorose. The AAA family (ATPases associated with a variety of cellular activities) and aquaglyceroporin genes were highly expressed in sophorose, whereas the ADP/ATP carrier genes were highly expressed in glucose (Additional file 6: Table S6). In addition, amino acids, oligopeptide, and ion transporter genes were identified as being regulated by the three carbon sources, with a larger number of genes expressed in cellulose.

### Deciphering the regulatory network of *T. reesei* in response to cellulose/sophorose

**Figure 5:**
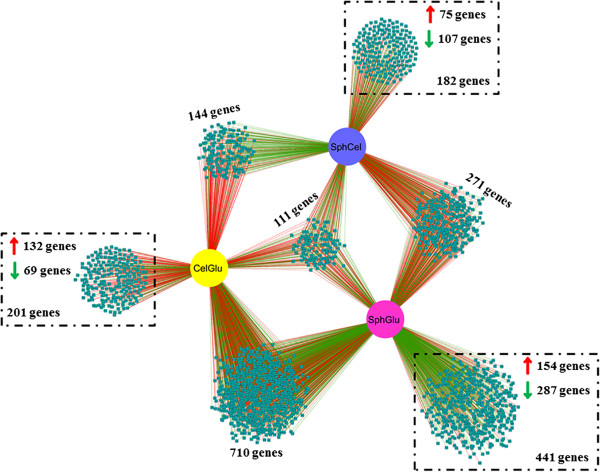
**Gene regulatory network (GRN) of 2,060 differentially expressed genes in *T. reesei *QM9414 in each tested condition.** Cellulose versus glucose (CelGlu), sophorose versus cellulose (SphCel) and sophorose versus glucose (SphGlu). Genes are represented as nodes (shown as squares), and interactions are represented as edges (shown as lines, that is, red indicates upregulated interactions and green indicates downregulated interactions), that connect the nodes: 3,385 interactions.

Using the experimental setup described above, we were able to identify a specific set of genes differentially regulated by the analyzed carbon sources. Using these data, the regulatory network of the genes identified as being modulated in a carbon source-dependent manner was reconstructed (Figure 5). Extensive overlapping between the differentially expressed genes in cellulose and sophorose (710 genes) was observed. Additionally, genes specifically associated with each condition were identified, as exemplified by the large number of genes (441) whose expression was specifically modulated during growth in sophorose compared to glucose. These genes represent either genes silenced in glucose but induced by sophorose (upregulated: 154 genes), or genes that are necessary for growth in glucose but dispensable for growth in sophorose (downregulated, 287 genes). Accordingly the analysis of genes specific to cellulose showed an over-representation of upregulated genes (132 genes) that are related to the expression of cellulase genes as compared to glucose (201 in total) (Figure 5).

The comparison between the two inducing conditions provided additional information by revealing new differentially expressed genes that were not identified via the comparison with glucose. From the 692 genes differentially expressed between cellulose and sophorose, only 75 and 107 genes were assigned specifically to sophorose or cellulose respectively (Figure 5).

**Figure 6:**
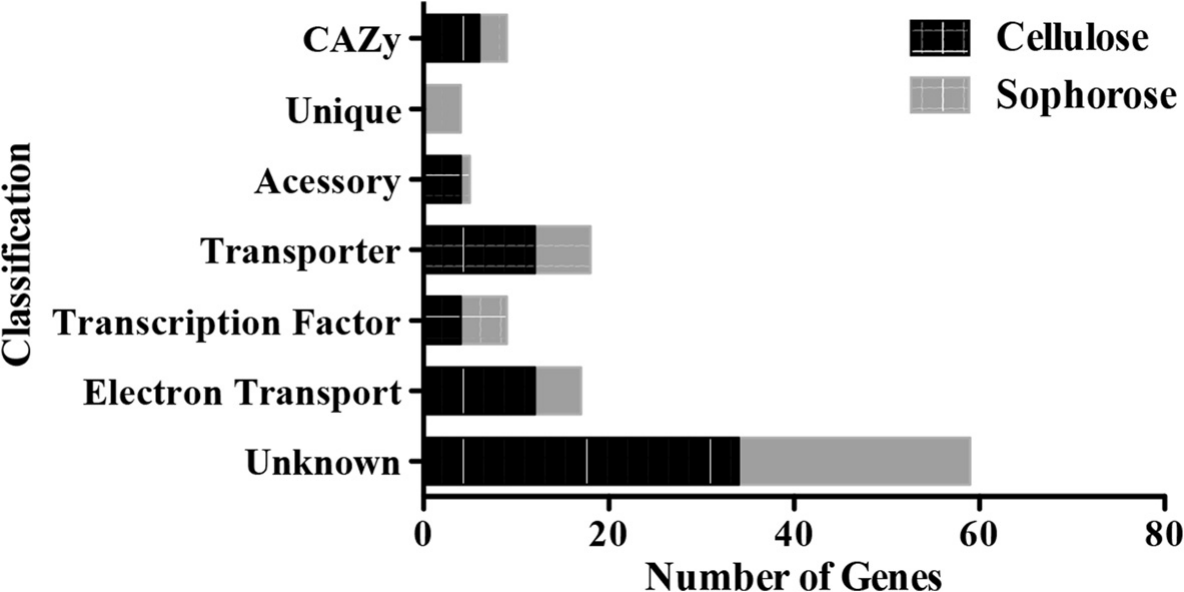
**Gene Ontology** (**GO) enrichment analysis of different classes of genes upregulated in cellulose and sophorose in *T. reseei.*** Significantly enriched categories (*P* ≤0.05) are shown. The complete list of differentially expressed genes is shown in Additional file 7: Table S7.

The majority of the genes identified from this network analysis were of unknown function. Importantly, a cellulose- or sophorose-specific enrichment of different gene classes was observed (Figure 6). During growth in cellulose, there was an enrichment of CAZy encoding genes (that is, GH64, GH 62, GH81, GH76, GH54), accessory proteins (Small secreted cysteine-rich protein (SSCRP), OOC1, and Epl1), transporters (most of them related to iron and metal transporters), TFs (*lae1*, C2H2 and Zn2Cys6 TFs) and a variety of proteins related to electron transport (Table S7). In contrast, there were only three CAZy encoding genes specific to growth in sophorose. However, four genes encoding *Trichoderma* species-specific proteins were only induced on sophorose, suggesting that *Trichoderma* possesses a specialized sophorose metabolism system (the complete list of differentially expressed genes is shown in Additional file 7: Table S7). The substantial overlap between the cellulose and sophorose transcriptomes supports the hypothesis that sophorose is a natural inducer of cellulase transcription, while the cellulose-specific enrichment for additional CAZymes and accessory proteins reflects the difficulty in the deconstruction of this insoluble substrate.

### Quantitative real-time PCR (RT-qPCR) analysis

**Figure 7:**
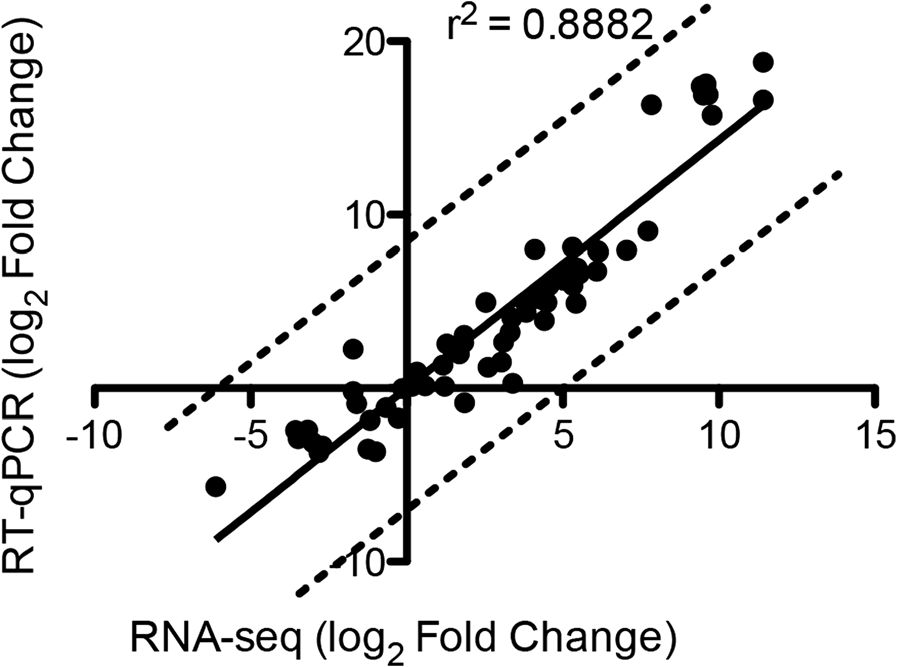
**Correlation between RNAseq and quantitative real-time PCR (RT-qPCR).** Comparison of log_2_ fold change of 20 genes obtained by RNA-seq and RT-qPCR. Real-time PCR was performed using the amplified cDNA from each RNA-seq sample. Strong, statistically significant Pearson correlation is shown between the expression levels measured using real-time PCR and RNA-seq.

The RNA-seq data were validated using 20 genes with mRNA accumulation that was modulated when the following comparisons were performed: cellulose versus glucose; sophorose versus cellulose; and sophorose versus glucose. The 10 upregulated genes were predominantly glycoside hydrolases and 10 downregulated genes were randomly chosen (see Additional file 8: Table S8). The log_2_ fold change in gene expression between the three comparisons obtained by RNA-seq and RT-qPCR demonstrated significant Pearson correlation (*r*^2^ = 0.8882), indicating the reliability of the RNA-seq analysis (Figure 7).

### Secretome analysis by two-dimensional DIGE

**Figure 8:**
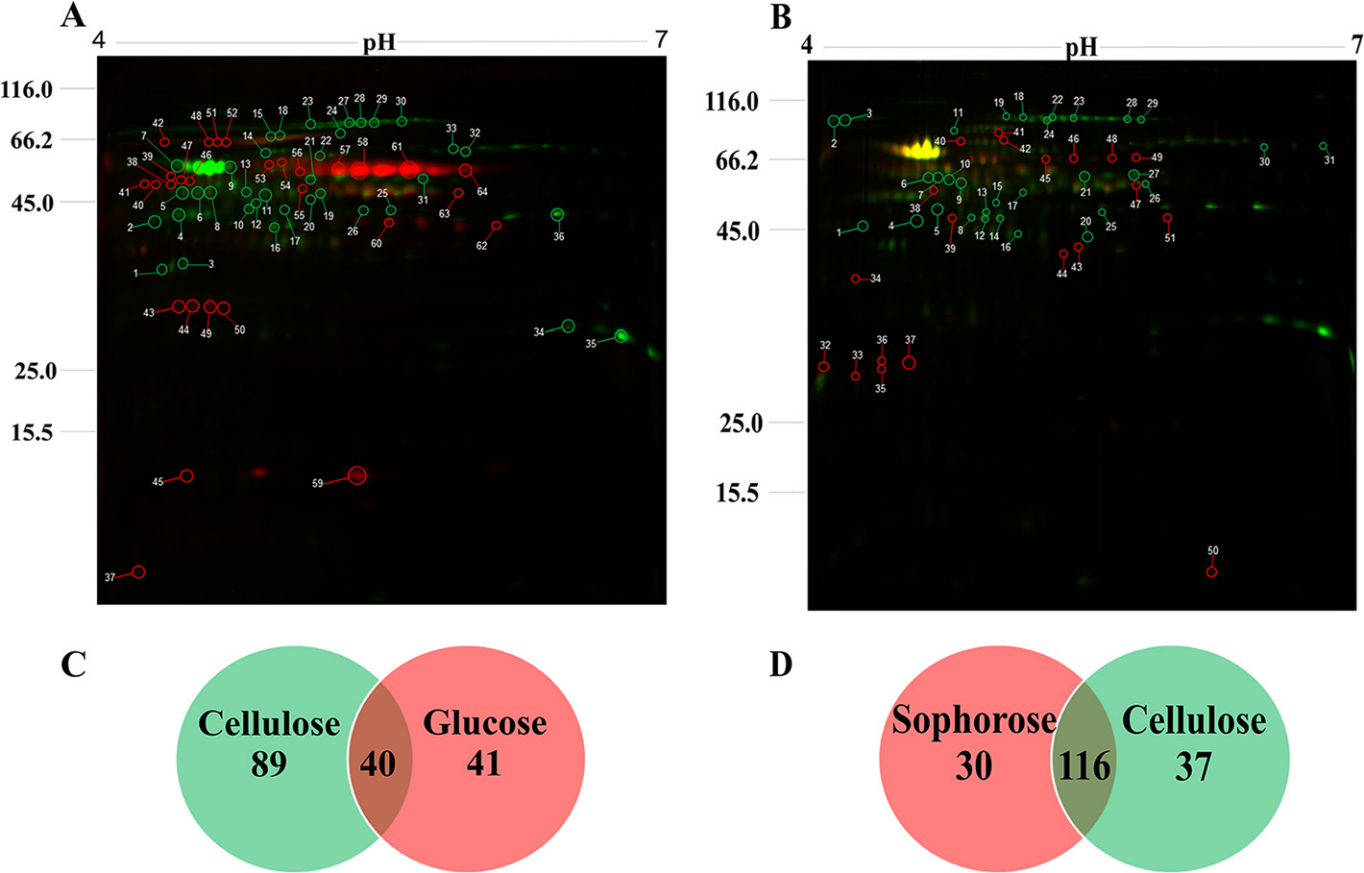
**Differential gel electrophoresis** (**DIGE) analysis of *T. reesei *secretome grown in different carbon sources. (A)** DIGE of cellulose (green spots) versus glucose (red spots). **(B)** DIGE of cellulose (green spots) versus sophorose (red spots). **(C)** Venn diagram from analysis of cellulose versus glucose. **(D)** Venn diagram from analysis of cellulose versus sophorose. The numbers in white indicate the spots subjected to liquid chromatography tandem mass spectrometry analysis. Protein IDs of identified spots are listed in Additional files 9 and 10: Table S.9.1 and S9.2).

The *T. reesei* secretome when grown in glucose, sophorose and cellulose were analyzed by quantitative proteomics (two-dimensional DIGE), followed by liquid chromatography tandem mass spectrometry (LC-MS/MS) analysis. The gels shown in Figure 8 are representative of all three independent gels and three biological replicates. The distribution of the spots indicates that most of the secreted proteins have isoelectric points <6.0 and a molecular weight >30 kDa. In some cases, the molecular weights and isoelectric points observed in the two-dimensional gels were higher than expected, probably due to post-translational changes. Another observation was that various different spots were assigned to the same protein, suggesting the presence of a number of isoforms or possibly degraded forms of the protein (Table S9.1 and S9.2, see Additional file 9 and Additional file 10, respectively).

The comparative analysis between the cellulose and glucose secretomes showed a total of 170 spots automatically detected by the software PDQuest (BioRad). Among these, 130 spots were statistically validated using a differential abundance ratio ≥2.0-fold (*P* ≤0.05). In total 89 spots were exclusively expressed in cellulose (Figure 8C) however, only 36 spots could be identified by mass spectrometry (MS) (Table S9.1). The identified proteins included classical cellulases (CEL7A and CEL6A), such as β-1-3-glucanosyltransferase, α-L-arabinofuranosidase, β-1,3-glucanase, α-1,2-mannosidase, Xylanase 4, β-xylosidase and isoforms, as well as a non-hydrolytic CIPI (Cellulose-induced protein). Furthermore, some proteases were also identified as being expressed during growth in cellulose, as well as an protein of unknown function (Trire2_55887) and a novel protein isoamyl alcohol oxidase (Trire2_73631), (see Additional file 9: Table S9.1). On the other hand, 41 spots were exclusively expressed during growth in glucose (Figure 8C). Among them, 28 spots were identified by MS (Additional file 9: Table S9.1): an acid phosphatase-like protein, various isoforms of isoamyl alcohol oxidase, subtilisin-like protease PPRC1, cell-wall glucanosyltransferase, amidase, a cerato-platanin, Epl1/Sm1 as well an SSCRP, and a unique protein (Trire2_121136).

When the sophorose secretome was compared to cellulose, the software PDQuest detected 183 spots, with 30 spots being exclusive to sophorose (≥2.0-fold; *P* ≤0.05), 37 exclusive to cellulose (≥2.0-fold; and *P* ≤0.05), and 116 spots common between the two conditions (Figure 8D). After MS analysis 20 spots from sophorose and 31 spots from cellulose were identified (see Additional file 10: Table S9.2). Among the enzymes classified as glycoside hydrolases, families GH3 (BXL1 β-xylosidase, β-glucosidase BGL1/CEL3a), GH64 (endo-β-1,3-glucanase), GH7 (Cellobiohydrolase CBH1/CEL7a, Endo-β-1,4-glucanase EGL1/CEL7b), GH72 (β-1-3-glucanosyltransferase), GH17 (glucan endo-1,3-β-glucosidase), GH28 (exo-rhamnogalacturonase RGX1), GH61 (polysaccharide monooxygenase CEL61a), GH74 (Xyloglucanase CEL74a), and GH30 (endo-β-1,4-xylanase XYN4) were found to be exclusive in cellulose cultures, whereas only the GH13 (α-amylase) was detected in sophorose. Furthermore, a larger number of proteins not related to glycoside hydrolase functions were detected in sophorose than in cellulose, such as SSCRP, lipoate-binding, ribonuclease T2, ubiquinol cytochrome reductase, DNAse, cell wall glucanosyltransferase, CIP1, ceramidase family protein, amidase, isoamyl alcohol oxidase, and ubiquitin fusion protein. Another interesting feature of the secretome was that both unknown proteins and *Trichoderma* species-specific proteins with described functions were found in both cellulose and sophorose, demonstrating the complexity and uniqueness of the *Trichoderma* secretome during cellulose degradation.

In order to correlate the gene expression data with the secretome, the fold change from cellulose versus glucose in both datasets was compared. There was 70.17% correlation between gene expression and secreted protein profiles (see Additional file 9: Table S9.1). When cellulose and sophorose cultures were compared, lower correlation was observed (47%), possibly due to many non-significant differences in expression (*P* <0.05) in RNAseq analysis (see Additional file 10: Table S9.2).

## Discussion

In natural environments, free-living organisms are continuously challenged with rapidly changing conditions that have a considerable impact on their lifestyle. Genomic and post-genomic techniques have revealed that free-living organisms dedicate a large percentage of their genes to sensing environmental signals and the subsequent coordination of gene expression in response to such cues. How the fungus *T. reesei* recognizes its substrate and activates the transcription of genes encoding transporters and TFs that culminate in the production of hydrolytic enzymes has been a subject of speculation since the 1960s. By using high-throughput genomic and proteomic approaches we describe both repressing (glucose) and de-repressing (cellulose or sophorose) conditions, identifying new players in cellulose degradation in *T. reesei*. In addition, the comparison between cellulose and sophorose, the hypothesized natural inducers of cellulase production, revealed a striking similarity in the global profiles.

The transcriptome study of *T. reesei* identified 123 genes that were specifically induced by cellulose, 154 by sophorose and 402 by glucose (Figure 3). Within these gene sets, 8 permease/transporter genes were induced in cellulose, 6 in sophorose and 11 in glucose (see Additional file 3: Table S3 and Additional file 8: Table S8 respectively). Of these 25 transporters, 10 showed possible homology to *N. crassa* homologues and one of them, MFS permease (Trire2_76800) (highly induced in the presence of cellulose), allowed *S. cerevisiae* to transport xylose [30,31]. Furthermore, a gene encoding a putative galactose permease (Trire2_62380) found in glucose and another MFS permease (Trire2_76800) regulated by cellulose, showed similarity to *S. cerevisiae* transporters involved in hexose uptake [26]. Additional transporters were induced by both cellulose and sophorose, suggesting that sophorose could be the natural inducer of cellulase gene transcription in *T. reesei*. Despite that, the functions of these transporters in *T. reesei* remain obscure. For instance, the transporter Trire2_3405 was recently identified to be specifically involved in cellulase induction by lactose [29], but has also been described as being involved in cellobiose transport [27]. Furthermore, the same transporter was upregulated during growth on wheat straw [22,28], cellulose or sophorose (Table 3). This lack of specificity by transporters could be explained by the close structure of cellobiose/lactose/sophorose or by the fact that some transporters can act as transporters and nutrient sensors. However, more detailed studies will be needed to characterize these transporters and generate a better understanding of the inducer/repressor transport system in *T. reesei.*

Global gene expression analysis by RNA-seq enabled the construction of gene regulatory networks (GRN) that enhanced the understanding of the interaction between different genes during the degradation and metabolism of cellulose. Studies on the control of catabolic genes related to the metabolism of simple substrates (such as glucose) performed in model organisms have revealed very complex GRN, thus, an even more sophisticated network controlling catabolic functions related to the metabolism of complex substrates, such as cellulose, could have been anticipated [32]. In the *T. reesei* model for cellulose degradation, the deep knowledge of the catabolic activities related to cellulose metabolism is accompanied by a very limited understanding of the regulatory pathways responsible for controlling gene expression [22,32,33]. In fact, despite the TFs, XYR1 and CRE1 [34,35], which regulate the induction or repression of the cellulolytic enzymes respectively, and a few more specific regulators (ACE1, ACE2, BGLR) [36,37] that have been experimentally characterized, there remains a lack of information on how, and to what extent, the expression of these enzymes are connected to the core GRN of *T. reesei*[32]. This is important as GRN in free-living organisms are usually densely connected and the final decision on the expression of a particular gene set is generally controlled by many different external/internal signals [38]. The collection of *omics* data provided here tries to fill this gap by providing a global analysis of *T. reesei* grown in three different substrates (cellulose, sophorose and glucose). From the analysis provided, we started building a bona fide regulatory network for this organism through the identification of 43 TF genes specifically induced in some particular growth conditions (see Additional file 5: Table S5). The GRN revealed that some of factors are exclusively induced in response to cellulose or sophorose (see Additional file 7: Table S7). For instance, the methyltransferase LAE1 has already been described as controlling the expression of cellulases, auxiliary factors for cellulose degradation, β-glucosidases and xylanases [39], proteins commonly found in response to inducers, cellulose, lactose and wheat straw [22,28]. However, our results showed that LAE1 is preferentially expressed in response to cellulose, indicating that the fungus has specific signaling for the metabolism of cellulose. This hypothesis is supported by the fact that recent study showed that LAE1 affects other components of cellulose degradation, such as non-ribosomal peptide synthases, ankyrin-repeat proteins, iron uptake, PTH11-receptors, and oxidases/monoxygenases [40], genes that were also upregulated in the presence of cellulose in our data and in the presence of wheat straw [22]. Another TF gene upregulated in response to cellulose (Trire2_120698) showed homology to the *Aspergillus nidulans* pH-responsive transcription factor *pacC.* It is known that this TF controls a range of functions in filamentous fungi [41]. Although studies have shown that pH is involved in cellulase production in *T. reesei*[42], the regulation of cellulase genes by any pH-responsive TF is still unknown.

The expression level of the *cre1* gene was low even in the presence of glucose. One explanation for this result is the fact that some TFs can act either directly on CAZyme encoding genes or indirectly by regulating other TFs that in turn regulate the expression of CAZyme genes. Here we identified some TF genes that are candidates for the indirectly transcriptional regulation, in a carbon source-dependent manner (see Additional file 5: Table S5). Some of these TFs could play an important role in the coordination of gene expression downstream in the network, either in association with the previously identified general factors at the target promoters or in isolation, in a sort of cascade signaling pathway. Additionally, the identified TFs could work as check points for the integration of different physiological/environmental signals, such as metabolic status of the cell, levels of light, presence of stresses, et cetera. [43,44]. The TFs identified here are candidates for further investigation into the mechanisms of signal integration in this biotechnologically relevant fungus. Understanding these missing regulatory interactions is pivotal for future attempts to synthetically engineer *T. reesei* for enhanced cellulolytic functions.

Analyses of the *T. reesei* secretome has commonly focused on growth in cellulose or lactose [45-47]. Besides the classical cellulases already described, our differential secretome showed the presence of polysaccharide monooxygenase, xyloglucanase CEL74a, and xylanases, induced by cellulose, whereas in sophorose, amidase, amylase and isoamyl alcohol oxidase they were described for the first time. The strong correlation between transcriptome and secretome data in the presented study is consistent with other comparable studies [29,48,49]. Furthermore, a comparison of the cellulose and sophorose transcriptome and differential secretome data did not detect a massive difference in any analyzed category of proteins. This observation suggest that the signaling for cellulose and sophorose to induce cellulase formation is very conserved and thus sophorose still remains a strong candidate as natural inducer.

Despite extensive work related to the regulation of cellulases in *T. reesei*, the real identity of the natural inducer is not yet established. New evidence has recognized cellobiose and cellodextrins as strong candidates for natural inducers [50]. Indeed, studies with *N. crassa*[51] and *A. niger*[52] have discredited sophorose as the natural inducer. It is known that *T. reesei* possesses a different mechanism for the regulation of cellulase production in response to sophorose when compared to other lignocellulose-degrading fungi [5]. Our GRN data showed little differences in the regulation of gene expression by the inducers cellulose and sophorose, suggesting that sophorose could be a natural cellulase inducer. But how did this divergence between *T. reesei* and other fungi occur? Comparative genomics between *T. atroviride, T. virens* and *T. reesei* suggest that the ancestral state of *Hypocrea*/*Trichoderma* was indeed a mycoparasitic, possibly of wood-degrading basidiomycetes [5]. *T. reesei* subsequently may have kept the mycoparasitic characteristic for substrate competition, converting cellobiose to sophorose by a transglycosylation reaction and then metabolizing sophorose. This hypothesis can be supported by the fact that new species-specific proteins were upregulated only in sophorose and by the fact that cellobiose and sophorose are transported and metabolized at different rates [50]. For this reason, we propose that both cellobiose and sophorose act as co-inducers of cellulase formation in *T. reesei*. These facts could explain why among lignocellulose-degrading fungi, *T. reesei* is the more efficient degrader, despite its smaller enzymatic arsenal.

## Conclusions

Our study shows little difference between gene expression and the secretome during the growth of *T. reesei* in cellulose and sophorose. The difference in gene expression is associated with CAZymes, accessory proteins, transporters, TFs, and electron transport. Together with recent literature, the results shown here suggest that both cellobiose and sophorose act as co-inducers of cellulase production in *T. reesei*. Further functional genomic investigations of the new players identified to be involved in growth in cellulose will open up new lines of research into clarifying cellulase and hemicellulase regulation in *T. reesei*. In addition, the data shown in this study will contribute to the construction of industrial strains of *T. reesei* that produce high levels of cellulase for plant cell-wall degradation thus facilitating its application in 2G-bioethanol production.

## Methods

### Strain and growth conditions

*T. reesei* strain QM9414 (ATCC 26921) was obtained from the Molecular Biotechnology Laboratory, Institute, TU Vienna, Austria. The strain was maintained on MEX medium (malt extract 3% (w/v) and agar-agar 2% (w/v)) at 4°C. QM9414 was grown on MEX medium at 28°C for 7 to 10 days to complete sporulation. For gene expression assays, a spore suspension containing approximately 10^7^ cells mL^-1^ was inoculated into 200 mL of Mandels-Andreotti medium [53] containing 1% (w/v) of cellulose (Avicel), or 2% (w/v) of glucose, or 1 mM of sophorose, as the sole carbon source. The cultures were incubated on an orbital shaker (200 rpm) at 28°C for 24, 48 and 72 hours using cellulose; for 24 and 48 hours with glucose; and 2, 4 and 6 hours with sophorose, as the carbon source. In the latter, the mycelium was previously grown on glycerol 1% (w/v) for 24 hours. After this time, the mycelium was washed with Mandels-Andreotti medium without peptone and then transferred to 20 mL of Mandels-Andreotti medium without peptone containing sophorose 1 mM. All experiments were performed in three biological replicates. The resulting mycelia were collected by filtration, frozen and stored at -80°C until RNA extraction and the supernatants were used for secretome analysis.

### RNA extraction

Total RNA was extracted from mycelia of each sample using TRIzol® RNA kit (Invitrogen Life Technologies, Carlsbad, CA, USA), according to the manufacturer’s instructions. RNAs concentrations were determined by spectrophotometric OD 260/280 and RNA integrity was verified by both the Agilent 2100 Bioanalyzer (Agilent Technologies, Waldbroon, Germany) and gel electrophoresis in 1% agarose.

### High-throughput sequencing (RNA-seq)

Total RNA of three biological replicates, cellulose (24, 48 and 72 hours), sophorose (2, 4 and 6 hours) and glucose (24 and 48 hours) were time points that were pooled, resulting in nine samples for the preparation of next-generation sequencing libraries using the TruSeq RNA Sample Prep kit (Illumina, San Diego, CA, USA). The total RNA samples obtained from *T. reesei* were lyophilized and stored using the RNAstable tube kit (Biomatrica, San Diego, CA, USA) in order to maintain the RNAs integrity for sequencing. Nine barcoded libraries (cel1-3, gluc 1–3 and soph 1–3) were prepared and sequenced by LGC Genomics GmbH (Berlin/Germany) using the Illumina Hiseq 2000 platform.

### Data analysis

The Illumina Hiseq 2000 system was used to sequence approximately 117 million 100 bp paired-end reads. These sequences were quality-filtered and mapped to the *Trichoderma reesei* 2.0 reference genome, available from the JGI Genome Portal (http://genome.jgi-psf.org/Trire2/Trire2.home.html), using the Bowtie aligner version 0.12.8 [54], allowing for two mismatches and only unique alignments. After alignment, Samtools version 0.1.18 [55] was used to process the alignments files, which were visualized using the Integrative Genomics Viewer [56]. The genes were annotated using *Trichoderma reesei* 2.0 reference genome and a local database provided by Professor CP Kubicek (TU, Vienna). Unknown proteins were defined as proteins that have yet to be assigned a function in any ascomycete and *T. reesei* species-specific proteins were defined to be proteins that did not occur in any other Pezizomycotina [57]. Bioconductor DESeq package version 1.10.1 [58] was utilized for the differential expression analysis, using two-fold change cutoff, that is, log_2_ fold change ≥1 or ≤ -1 and an adjusted *P*-value ≤0.05 as thresholds. Samples were normalized using median log deviation DESeq, available in the Bioconductor package. Cluster analysis was carried out using the software Mev v.4.6.1 to identify cellulose, sophorose and glucose regulons. The average linkage method was used for cluster generation, with uncentered correlation as the similarity metric. Functional enrichment analysis of differentially expressed genes was performed using GO terms was performed using the BayGO algorithm [59]. GO terms significantly enriched, (that is, with *P*-values ≤0.05) were analyzed further. Raw sequence data and count data for all samples are available at [GEO: GSE53629]. CAZy classification was performed based upon the re-annotation of CAZy genes of *T. reesei* according to Hakkinen *et al*. [9].

### Regulation network of *T. reesei*

In order to reconstruct the regulatory network of *T. reesei* under the experimental condition analyzed, a table using the following information was generated: inducing condition (QMCelGlu, QMSphCel and QMSphGlu, selecting differentially expressed genes, up- and downregulated in each condition, *P* ≤0.05), the interaction type (up- or downregulated) and the target gene (that is, the protein ID of each gene affected). This analysis provides a network representation for all the genes (2,060 in total) shown in the heat map of Figure 2. The regulatory network was then generated using the Cytoscape 3.0.1 software [60].

### Quantitative qRT-PCR analysis

Differentially expressed genes identified by the RNA-seq analysis were further analyzed by qRT-PCR in order to validate their expression. In this analysis, the same RNA samples, utilized for the RNA sequencing experiments were re-used. Approximately, 1 μg of RNA was treated with DNAseI (Thermo scientific) and reverse-transcribed to cDNA using the First Strand cDNA kit Maxima™ Synthesis according to manufacturer’s instructions. The cDNA was diluted to 1/50 fold and used for real-time PCR analysis in the Bio-Rad CFX96™ System, using SsoFast™EvaGreen®Supermix (Bio-Rad, San Francisco, CA, USA) for signal detection in accordance with the manufacturer’s instructions. Genes encoding actin (*act*) and a small GTPase SAR/ARF-type (*sar1*) were used as endogenous controls according to [61]. Twenty genes, including up- and downregulated genes in cellulose compared to glucose samples (see Additional file 11: Table S1), were used for qRT-PCR analysis. The following amplification reaction was used: 95°C for 10 minutes followed by 39 cycles of 95°C for 10 seconds, 60°C for 30 seconds followed by a dissociation curve of 60°C to 95°C with an increment of 0.5°C for 10 seconds. Gene expression values were calculated according to the 2^-ΔΔCT^ method [62] using the QM9414 strain growth on glucose as the reference sample. Data analysis was performed using GraphPad Prism v 5.1 software.

### Sample preparation for proteomic analysis

The protein concentration was determined using the kit Bio-Rad Protein Assay, based on the Bradford method. Protein concentration was adjusted to 1 μg /μl, and 150 μg used for in two-dimensional DIGE and 300 μg in two-dimensional SDS-PAGE. Samples were precipitated using 10% tricarboxylic acid (TCA) in acetone and incubated at -20°C overnight. Samples were centrifuged at 10,000 g for 10 minutes at 4°C and the supernatant removed. β-mercaptanol was added (0.07%) in acetone and centrifuged at 10,000 g for 10 minutes at 4°C. This was repeated three times, discarding each supernatant after centrifugation. After precipitation, the pellet was purified using Ettan2D Clean-Up Kit (GE Healthcare, Waukesha, WI, USA).

### Two-dimensional differential gel electrophoresis

The proteins (150 μg) secreted by *T. reesei* under different conditions were labeled with 400 pmol CyDyes (Cy3 or Cy5) according to the manufacturer’s instructions (GE Healthcare, Waukesha, WI, USA). An internal pool generated by equal amounts of all samples was labeled with Cy2. The isoelectric focusing was carried out on 18-cm linear IPG strips, pH 4–7, with the addition of 1.2% DeStreak and 1% IPG buffer 4–7 (GE Healthcare). Isoelectric focusing was performed on IPGphor III in four steps: 500 V for 60 minutes, 1000 V for 60 minutes, 8000 V for four hours and 8000 V for six hours. The strips were reduced (1.5% w/v dithioerythritol) and alkylated (2.5% w/v iodocetamide) in equilibration buffer (6 M urea, 50 mM Tris–HCl, pH 6.8, 30% glycerol, 2% SDS). Equilibrated strips were run on homogeneous 12.5% polyacrylamide gels using an Ettan DALTsix electrophoresis (GE Healthcare). All the experiments resulted in three independent replicates for each experimental condition. The preparative gels were stained using colloidal Coomassie and destained with Milli-Q water to remove excess Coomassie particles. Gels were scanned using the laser scanner Pharos FX Plus (Bio-Rad) and Quantite One software (Bio-Rad) using a resolution of 100 μm and the appropriate wavelength. The images were analyzed with the software PDQuest Advanced 2-D Analysis Software (Bio-Rad). Differential expression was determined by statistical analyses using the *t*-test, as the parameter of significance (*P* ≤0.05).

### Protein identification by mass spectrometry

Spots which increased or decreased in volume (protein content) by two-fold or more were manually excised from the gels and washed four times with 50 mM NH_4_HCO_3_ containing 50% v/v acetonitrile (ACN) to remove SDS and dye. They were then washed with ACN and completely dried in a SpeedVac (Savant Instrument, Farmingdale, NY, USA). Each spot was rehydrated with 20 μl 50 mM NH_4_HCO_3_ containing 0.3 μg of sequencing grade modified trypsin (Promega, Madison, WI, USA). After 30 minutes of rehydration with the trypsin solution, spots were covered with 50 mM NH_4_HCO_3_. The hydrolysis reaction was carried out at 37°C for 24 hours and stopped by the addition of 10 μl formic acid 1%. Peptides were extracted twice from the gel with 40 μl 0.1% v/v formic acid solution containing 50% v/v ACN for 1 hour. Extracts were dried in a SpeedVac and resuspended in 35 μl 0.1% v/v formic acid solution containing 5% v/v ACN for MS injection. Samples were then analyzed in an XEVO-TQS mass spectrometer (Waters) coupled with a UPLC chromatography system (Waters). Liquid chromatography separation was performed in a 15 cm column (ACQUITY UPLC HSS C18, 100 Å, 1.8 μm, 1 mm × 150 mm, Waters) using a 30-minute linear gradient from 5 to 30% of ACN in 0.1% formic acid at 150 μl/minute. The spectra were acquired in a data-dependent mode in an m/z range of 400 to 1,500, with selection of the two most abundant ions of each MS spectrum for MS/MS analysis. MS parameters were as follows: capillary voltage of 3.5 KV and capillary temperature of 400°C. Acquired raw data were converted to mzXML and automatically processed by an in-house installation of Labkey Server v12, using theX!Tandem search algorithm [63]. The minimum criterion for peptide matching was performed using a Peptide Prophet [64] score greater than 0.8. Peptides that met these criteria were further grouped to protein sequences using the Protein Prophet [65] algorithm and only proteins with an error rate of 5% or less and two peptides sequences identified were considered as valid identifications.

## Abbreviations

2G: second-generation; AA9: Auxiliary family activity 9; ABC: ATP binding cassette; ABF: arabinofuranosidase; ACN: acetonitrile; AXE: acetyl xylan esterase; bp: base pairs; CAZy: carbohydrate active enzyme; CE: carbohydrate esterase; CIP: Cellulose-induced protein; DIGE: differential gel electrophoresis; FC: fold-change; FPKM: fragments per kilobase of exon per million fragments mapped; GH: glycoside hydrolase; GH31: α-xylosidase; GRN: gene regulatory network; LC/MS/MS: liquid chromatography tandem mass spectrometry; MEX: malt extract medium; MFS: Major facilitator superfamily; MS: mass spectrometry; PMO: polysaccharide monooxygenase; RT-qPCR: quantitative real-time PCR; SSCRP: Small secreted cysteine-rich protein; SWOI: swollenin; TF: transcriptional factor; xylanase: xyn.

## Competing interests

The authors declare that there are no competing interests.

## Authors’ contributions

LSC performed the experimental design, laboratory experiments, performed the bioinformatics analysis, and drafted the manuscript. WRP performed the experimental design, DIGE experiments, and drafted the manuscript. ACCA performed the bioinformatics analysis, and drafted the manuscript. ASS performed the bioinformatics analysis, drafted the manuscript, and interpreted the data for the work. RS-R, NMM-R, AR performed the bioinformatics analysis, and drafted the manuscript. NB and GHG drafted and revised the manuscript and discussion, and interpreted the data for the work. VMF performed the secretome analysis and drafted the manuscript. GFP supervised the bioinformatics analysis and drafted the manuscript. RNS designed the project, supervised the research study, prepared/drafted the manuscript, and final approved of the version to be published. All the authors have read and approved the final manuscript.

## Supplementary Material

Additional file 1: Table S2 Summary RNA-seq reads obtained (Illumina Hiseq 2000) in this study. Click here for file

Additional file 2: Figure S1 Biological replicates used for the RNA-seq analysis. **(A)** Graphs representing the Pearson correlation between biological replicates of each sample. **(B)** Principal component analysis (PCA) of the samples analyzed. **(C)** boxplot of all normalized samples and **(D)** boxplots of raw data. Click here for file

Additional file 3: Table S3.1 Genes from cellulose regulon. **Table S3.2.** Genes from glucose regulon. **Table S3.3.** Genes from sophorose regulon protein. Click here for file

Additional file 4: Table S4A Carbohydrate active enzyme (CAZy) genes that are upregulated in sophorose. **Table S4B.** CAZy enzymes that are upregulated in celulose. **Table S4C.** CAZy enzymes that are upregulated in glucose. Click here for file

Additional file 5: Table S5 The main transcription factors genes induced in presence of cellulose, sophorose and glucose. Click here for file

Additional file 6: Table S6 Upregulated transporters genes in presence of cellulose, sophorose and glucose. Values are expressed in log2 fold change. Click here for file

Additional file 7: Table S7 Differentially expressed genes in cellulose and sophorose. Numbers are expressed as log_2_ fold change. Click here for file

Additional file 8: Table S8 Comparison of the gene expression levels assayed by RNA-seq and RT-qPCR. The numbers highlighted in red did not correlate. Click here for file

Additional file 9: Table S9 Identified proteins from differential gel electrophoresis (DIGE) analysis between cellulose and glucose. Click here for file

Additional file 10: Table S10 Identified proteins from differential gel electrophoresis (DIGE) analysis between cellulose and sophorose. Click here for file

Additional file 11: Table S1 Primers used in the validation of differentially expressed genes. Click here for file
