# Supplementary material for: Comparative metabolism of cellulose, sophorose and glucose in Trichoderma reesei using high-throughput genomic and proteomic analyses
Source: Biotechnol Biofuels. 2014 Mar 21;7:41. doi: 10.1186/1754-6834-7-41 (PMC3998047; doi:10.1186/1754-6834-7-41)
Supplement: Additional file 1: Table S2 — Summary RNA-seq reads obtained (Illumina Hiseq 2000) in this study. [file 1754-6834-7-41-S1.pdf]

Table S2. Summary RNA-seq reads obtained (Illumina HiSeq 2000) in this study.

Samples	Raw Reads	Quality Reads	Mapped Reads	% Mapped Reads	Number of nucleotides (GB)
QM9414 Cel1	28483604	24284506	16951434	69.80	3.39
QM9414 Cel2	16036616	14420917	10172333	70.54	2.03
QM9414 Cel3	19224967	17304525	11683287	67.52	2.34
QM9414 Sph1	14311488	11341066	8445229	68.83	1.69
QM9414 Sph2	17782393	33172592	11041736	68.34	2.21
QM9414 Sph3	19691061	23138683	12645886	70.86	2.53
QM9414 Glu1	13341102	12269998	7814715	68.91	1.56
QM9414 Glu2	36490949	16157814	22484949	67.78	4.50
QM9414 Glu3	25600303	17845732	15366090	66.41	3.07
Total	190962483	169935833	116605659	68.78	23.32
