# Supplementary material for: Comparative metabolism of cellulose, sophorose and glucose in Trichoderma reesei using high-throughput genomic and proteomic analyses
Source: Biotechnol Biofuels. 2014 Mar 21;7:41. doi: 10.1186/1754-6834-7-41 (PMC3998047; doi:10.1186/1754-6834-7-41)
Supplement: Additional file 2: Figure S1 — Biological replicates used for the RNA-seq analysis. (A) Graphs representing the Pearson correlation between biological replicates of each sample. (B) Principal component analysis (PCA) of the samples analyzed. (C) boxplot of all normalized samples and (D) boxplots of raw data. [file 1754-6834-7-41-S2.pdf]

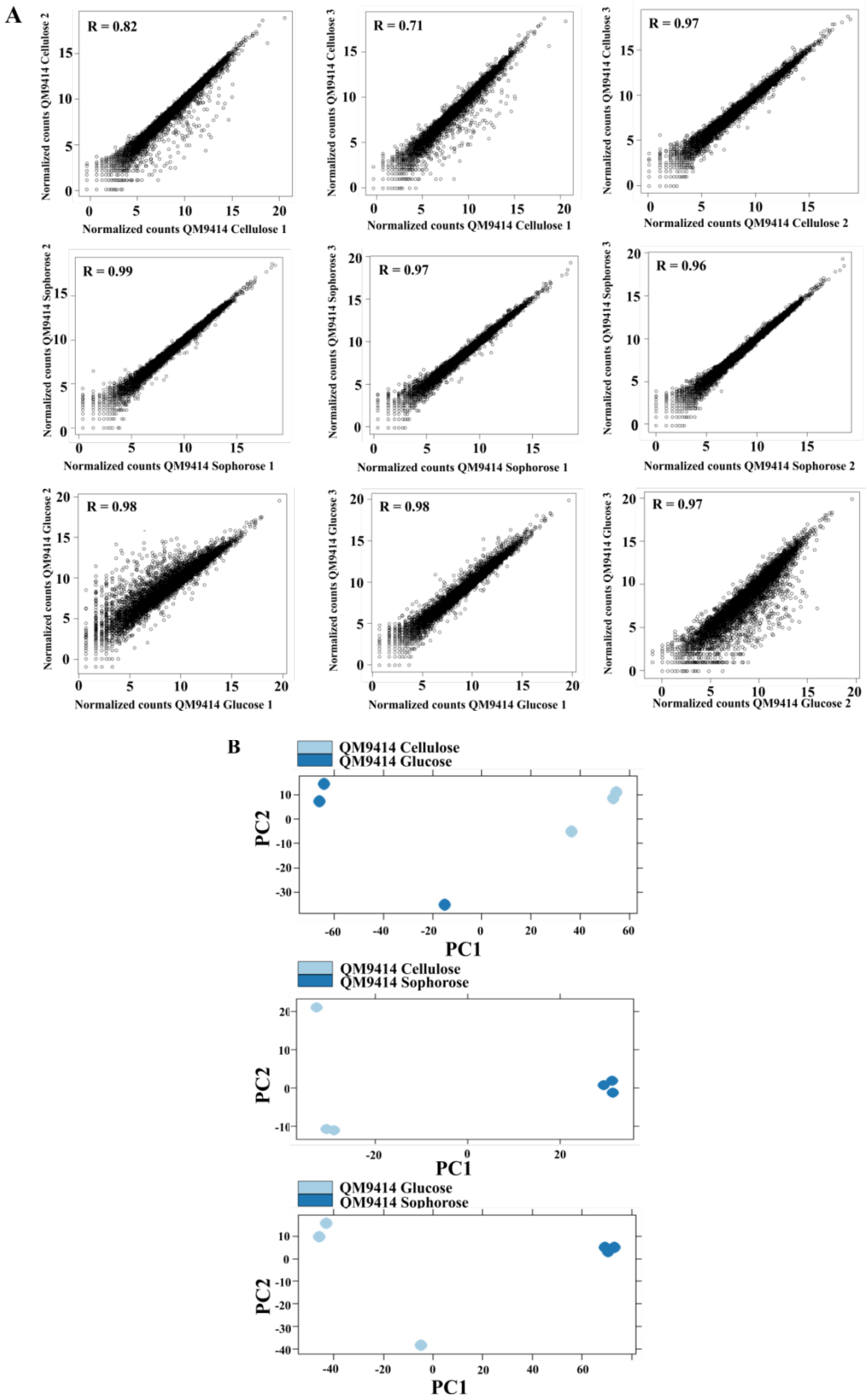
A
R = 0.82
Normalized counts QM9414 Cellulose 2
Normalized counts QM9414 Cellulose 1
R = 0.71
Normalized counts QM9414 Cellulose 3
Normalized counts QM9414 Cellulose 1
R = 0.97
Normalized counts QM9414 Cellulose 3
Normalized counts QM9414 Cellulose 2
R = 0.99
Normalized counts QM9414 Sophorose 2
Normalized counts QM9414 Sophorose 1
R = 0.97
Normalized counts QM9414 Sophorose 3
Normalized counts QM9414 Sophorose 1
R = 0.96
Normalized counts QM9414 Sophorose 3
Normalized counts QM9414 Sophorose 2
R = 0.98
Normalized counts QM9414 Glucose 2
Normalized counts QM9414 Glucose 1
R = 0.98
Normalized counts QM9414 Glucose 3
Normalized counts QM9414 Glucose 1
R = 0.97
Normalized counts QM9414 Glucose 3
Normalized counts QM9414 Glucose 2
B
QM9414 Cellulose
QM9414 Glucose
PC2
PC1
QM9414 Cellulose
QM9414 Sophorose
PC2
PC1
QM9414 Glucose
QM9414 Sophorose
PC2
PC1

C

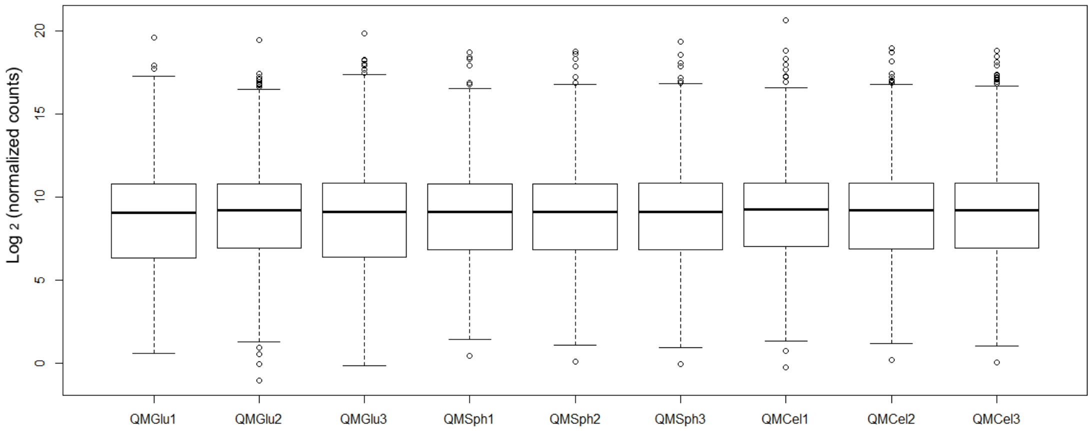

D

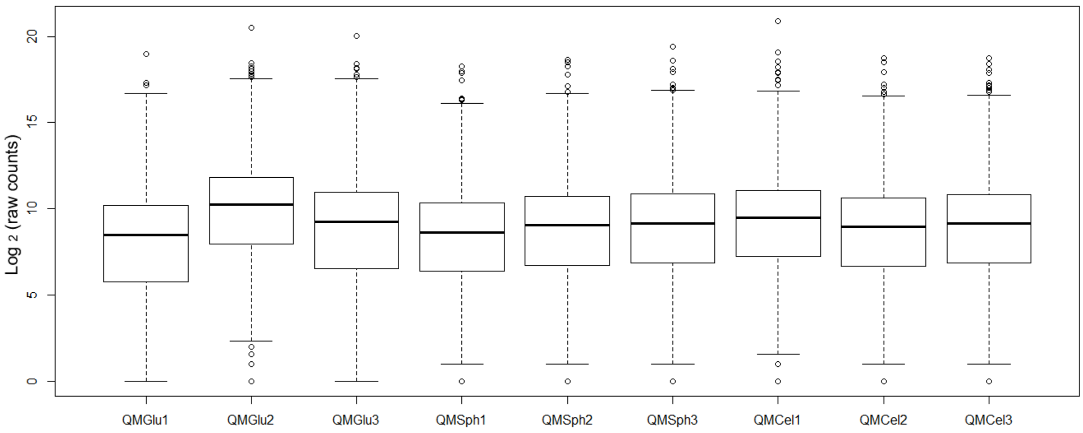

Fig. S1. Biological replicates used for the RNA-seq analysis. (A) Graphs representing the Pearson correlation between biological replicates of each sample. A high Pearson correlation was obtained demonstrating the reliability of RNA-seq analysis ($r^2 \geq 0.71$). (B) Principal component analysis (PCA) of the samples analyzed. (C) boxplot of all normalized samples and (D) boxplots of raw data, showing that all samples and conditions are comparable.
