# Supplementary material for: Comparative metabolism of cellulose, sophorose and glucose in Trichoderma reesei using high-throughput genomic and proteomic analyses
Source: Biotechnol Biofuels. 2014 Mar 21;7:41. doi: 10.1186/1754-6834-7-41 (PMC3998047; doi:10.1186/1754-6834-7-41)
Supplement: Additional file 3: Table S3.1 — Genes from cellulose regulon. Table S3.2. Genes from glucose regulon. Table S3.3. Genes from sophorose regulon protein. [file 1754-6834-7-41-S3.pdf]

Table S3.1. Genes from cellulose regulon

protein id	qmCelGlu	qmSphCel	qmSphGlu	GOTerm	Description
69957	11.00124	-3.67292	0	cellular_component	MFS permease
56996	10.84896	-5.4028	0	biological_process	GH5 β-Mannanase MAN1
69944	10.4997	-2.00703	0	biological_process	GH31 α-xylosidase/α-glucosidase
73632	8.060113	-1.02455	0	molecular_function	CE5 acetyl xylan esterase AXE1
111849	7.847975	-2.05627	0	biological_process	GH30 endo-β-1,4-xylanase XYN4
66788	6.462193	-2.65539	0	#N/D	unknown protein
81087	6.398736	-1.28376	0	biological_process	aminopeptidase Y
119857	5.424444	-4.85149	0	#N/D	unique protein
55887	4.996584	-1.07843	0	#N/D	unknown protein, secreted
124259	4.930999	-5.10259	0	#N/D	SSCRP
103039	4.719794	-3.63993	0	molecular_function	peptidase S41
112258	4.665277	-8.0687	0	#N/D	unknown protein
123955	4.53182	-4.22405	0	#N/D	Epl1/Sm1
72379	4.39949	-6.11659	0	molecular_function	conidiospore surface protein cmp1
64125	4.320885	-5.9993	0	biological_process	Serine/threonine protein kinase
103012	4.280628	-2.47662	0	molecular_function	taurine catabolism dioxygenase, alpha-ketoglutarate dependent
62166	4.238351	-3.46815	0	biological_process	GH2 β-mannosidase
76682	4.17135	-5.42135	0	molecular_function	PDR-type ABC transporters
108586	4.169976	-1.31347	0	#N/D	unknown protein
106879	4.126278	-4.89691	0	#N/D	unknown protein, secreted
55886	4.086267	-7.06701	0	#N/D	GH16 glucan endo-1,3(4)-β-D-glucosidase
122975	4.061222	-6.12989	0	biological_process	unique protein with collagen triple helix repeat
103537	3.955284	-2.30952	0	molecular_function	GCN5-N-acetyltransferase
111915	3.928892	-1.91763	0	#N/D	SSCRP

119552	3.884462	-7.08968	0	#N/D	unique protein
121004	3.823238	-2.95485	0	#N/D	unknown protein
74282	3.79437	-4.69239	0	molecular_function	QI74 orthologue
124079	3.78744	-7.76244	0	molecular_function	Multicopper oxidases
123199	3.776738	-2.96214	0	#N/D	SSCRP
80058	3.709685	-1.70919	0	cellular_component	MFS permease
55306	3.690156	-3.36522	0	biological_process	Acyl-CoA synthetase
124002	3.681603	-2.81626	0	#N/D	Sexual differentiation process protein ISP4
56911	3.658973	-3.79584	0	cellular_component	Urea transporter
108642	3.643899	-8.62071	0	#N/D	unknown protein
124277	3.632144	-5.26032	0	#N/D	SSCRP
107960	3.535617	-4.71033	0	#N/D	unique protein with WSC domain
60116	3.483245	-1.86889	0	molecular_function	MRP-type ABC transporter
76800	3.452257	-2.36051	0	cellular_component	MFS permease
70803	3.441678	-3.66159	0	biological_process	bifunctional catalase/peroxidase
52476	3.436203	-3.97873	0	#N/D	unknown protein
123723	3.337524	-1.49514	0	#N/D	Arylacetamide deacetylase
106538	3.32656	-4.76127	0	biological_process	HFB4
57204	3.158939	-5.73772	0	#N/D	lipase, secreted
56499	3.09121	-2.5582	0	#N/D	N-acyl-phosphatidylethanolamine-hydrolyzing phospholipase D, putative
104081	3.050274	-3.44921	0	#N/D	coenzyme F420-dependent N5,N10-methylene tetrahydromethanopterin reductase
82208	3.045582	-6.74182	0	biological_process	PKS
120697	3.030373	-1.32932	0	#N/D	SSCRP
65162	3.017136	-1.09172	0	molecular_function	GH18 endo-N-acetyl-ß-D-glucosaminidase Endo T
111990	2.943588	-1.63072	0	molecular_function	unknown protein
67472	2.916887	-2.18931	0	#N/D	unknown protein
61642	2.883611	-1.57298	0	biological_process	unknown protein, secreted
105269	2.836028	-1.98511	0	molecular_function	Zn2Cys6 transcriptional regulator

34252	2.801598	-2.56484	0	#N/D	unknown protein
74807	2.796392	-2.72018	0	biological_process	GH76 GPI-anchored α-1,6-mannanase
63413	2.69473	-1.13564	0	#N/D	unknown protein, only in ascomycota
123236	2.666417	-7.71334	0	#N/D	SSCRP
123226	2.643237	-1.09443	0	biological_process	GH37 α,α-trehalase
68122	2.598386	-2.75856	0	cellular_component	MFS permease
4430	2.562177	-3.421	0	molecular_function	Developmental regulatory protein WetA
108649	2.536881	-1.52767	0	#N/D	unknown protein
107680	2.525946	-1.65912	0	biological_process	DNA photolyase, class I, rapidly regulated by blue light in T. harzianum
122579	2.339876	-1.93956	0	#N/D	unknown protein, 4TM
123979	2.323299	-3.86245	0	molecular_function	peptide transporter MTD1
75165	2.296581	-2.4059	0	cellular_component	MFS permease
122091	2.295719	-1.309	0	molecular_function	phospholipase of papatin-family
37665	2.26766	-2.17915	0	#N/D	unknown protein
103487	2.204707	-1.81207	0	#N/D	glutathione S transferase
74953	2.197302	-2.73486	0	cellular_component	MFS permease
68204	2.182606	-1.34245	0	biological_process	NRPS
123611	2.148425	-1.01863	0	cellular_component	amino acid permease
47127	2.145926	-1.30345	0	biological_process	peptidase M18
111912	2.143523	-3.79662	0	biological_process	carbonic anhydrase
3049	2.137738	-2.19841	0	molecular_function	methionine aminopeptidase
63919	2.132146	-2.59246	0	biological_process	Cys/Met metabolism PLP-dependent enzyme
82026	2.048632	-2.96212	0	molecular_function	trans-aconitate methyltransferase
79644	2.043838	-1.33158	0	cellular_component	metal ion transporter SMF2
110910	2.017442	-2.61674	0	molecular_function	subtilisin like protease
122095	1.969711	-1.21545	0	#N/D	unknown protein, secreted, only present in ascomycota
81442	1.966209	-1.18305	0	cellular_component	Amino acid transporters
49970	1.927587	-1.25915	0	cellular_component	H+ nucleoside cotransporter

75027	1.90531	-1.0691	0	#N/D	unknown protein
106686	1.90492	-1.62023	0	#N/D	unknown protein
55443	1.896288	-1.88015	0	#N/D	unknown protein
69972	1.85073	-1.07398	0	molecular_function	Zn2Cys6 transcriptional regulator
108781	1.813643	-1.6293	0	#N/D	xenobiotic compound monoounknown proteingenase, DszA family
119956	1.764052	-1.31229	0	#N/D	unknown protein
121164	1.753883	-1.186	0	molecular_function	Zn2Cys6 transcriptional regulator
55668	1.753168	-1.19113	0	#N/D	unknown protein
119616	1.742086	-1.33433	0	biological_process	Serine/threonine protein kinase
121074	1.738467	-1.24994	0	biological_process	homeobox transcriptional regulator
66583	1.708849	-1.29632	0	#N/D	unknown protein
124283	1.696034	-1.57457	0	#N/D	unknown protein, SET and MYND domains
108775	1.695056	-1.76543	0	biological_process	transcription factor AbaA
112567	1.682164	-2.92016	0	molecular_function	taurine catabolism dioxygenase
67133	1.64134	-1.45754	0	#N/D	unknown protein
79361	1.633439	-1.07384	0	#N/D	unknown protein
22388	1.605153	-1.39709	0	#N/D	unknown protein
119896	1.584839	-1.12271	0	biological_process	malic enzyme
79329	1.583101	-1.06945	0	cellular_component	MFS permease
23228	1.570298	-1.59412	0	#N/D	unknown protein Duf1479
49112	1.55378	-3.84609	0	molecular_function	Glutamate-1-semialdehyde aminotransferase
49753	1.52048	-1.44871	0	molecular_function	L-arabinitol 4-dehydrogenase
123608	1.502312	-1.90673	0	#N/D	unique protein
2735	1.472846	-1.09992	0	molecular_function	GH18 chitinase CHI18-6
5319	1.417932	-2.00327	0	#N/D	unknown protein
55351	1.400736	-1.66605	0	#N/D	Alkyl hydroperoxide reductase/peroxiredoxin
45717	1.367745	-1.54593	0	cellular_component	GH47 α-1,2-mannosidase
5011	1.341624	-1.84306	0	#N/D	SSCRP

68254	1.334731	-1.50779	0	molecular_function	Zn2Cys6 transcriptional regulator
82374	1.312784	-1.21245	0	#N/D	unknown protein
121082	1.28424	-2.15774	0	#N/D	unique protein
123114	1.27733	-1.33755	0	molecular_function	Heat shock protein 90
107055	1.266147	-1.39088	0	#N/D	unknown protein
58561	1.263827	-1.98606	0	cellular_component	MFS permease
110007	1.224278	-1.64936	0	#N/D	unique protein
121653	1.188334	-4.50715	0	#N/D	unknown protein
120415	1.153904	-1.94464	0	#N/D	unknown protein
5502	1.14394	-1.2792	0	biological_process	unknown protein
57088	1.113523	-1.48591	0	cellular_component	Sulfate/bicarbonate/oxalate exchanger SAT-1 and related transporters (SLC26 family)
58790	1.102951	-1.06294	0	molecular_function	glycerol-3-phosphate phosphatase, putative
121163	1.024147	-2.32382	0	#N/D	unknown protein
65039	1.020766	-1.60161	0	#N/D	sexual development protein
121098	1.002483	-1.65021	0	molecular_function	Multicopper oxidases

Table S3.2. Genes from glucose regulon.

protein id	qmCelGlu	qmSphCel	qmSphGlu	GOTerm	Description
79816	-5.79292	0	-9.35374	molecular_function	unknown protein; secreted
73173	-5.7447	0	-6.83766	biological_process	HFB1
122824	-5.47299	0	-6.74858	#N/D	PTH11 GPCR
73631	-5.31654	0	-4.24494	biological_process	isoamyl alcohol oxidase
110316	-5.18038	0	-5.47377	cellular_component	Amino acid transporters
73623	-4.98577	0	-6.30765	biological_process	flavoprotein monooxygenase
69115	-4.82674	0	-7.49556	#N/D	dienelactone hydrolase
73618	-4.82593	0	-4.91569	molecular_function	PKS
73621	-4.70126	0	-4.74696	molecular_function	PKS
123084	-4.58731	0	-8.36915	molecular_function	chloroperoxidase
76641	-4.45446	0	-7.30874	cellular_component	MFS permease
81525	-4.43039	0	-7.44808	molecular_function	isoflavone reductase
112499	-4.39842	0	-6.81835	molecular_function	Zn2Cys6 transcriptional regulator
102499	-4.38538	0	-3.33779	molecular_function	Zn2Cys6 transcriptional regulator
105242	-4.36788	0	-6.42209	molecular_function	SAM-dependent methyltransferases
70520	-4.33338	0	-9.26312	molecular_function	short chain dehydrogenase/reductase
104106	-4.32043	0	-7.02046	cellular_component	ADP/ATP carrier protein
62522	-4.25783	0	-5.51784	#N/D	SSCRP
43701	-4.16861	0	-4.77422	cellular_component	MFS multidrug transporter
69956	-4.11358	0	-4.93266	molecular_function	Alcohol dehydrogenase, class V
23382	-4.02797	0	-9.21302	molecular_function	aldehyde reductase AKR7
80879	-3.90724	0	-4.38137	cellular_component	MFS H+/oligopeptide transporter
102497	-3.87955	0	-3.70265	molecular_function	Zn2Cys6 transcriptional regulator

62359	-3.82127	0	-7.05021	molecular_function	isoflavon reductase
111818	-3.80028	0	-6.09161	biological_process	aspartyl protease
53503	-3.75446	0	-6.48232	cellular_component	unknown protein
30759	-3.68413	0	-8.50801	molecular_function	zinc containing alcohol dehydrogenase superfamily
22453	-3.68194	0	-5.35585	#N/D	glutathione-S-transferase
122998	-3.63987	0	-7.63734	#N/D	unknown protein
49274	-3.63002	0	-4.45765	biological_process	glucan endo-1,3(4)-β-D-glucosidase
23240	-3.47224	0	-6.03781	#N/D	unknown protein
69696	-3.46886	0	-6.29133	#N/D	coenzyme F420-dependent N5,N10-methylene tetrahydromethanopterin reductase
123468	-3.4377	0	-7.06034	biological_process	IMP dehydrogenase
64920	-3.42556	0	-4.85427	cellular_component	Monocarboxylate transporter
56838	-3.41262	0	-3.18808	molecular_function	short chain dehydrogenase/reductase
121620	-3.34577	0	-2.48887	#N/D	NMT1 thiamine biosynthesis protein
109394	-3.22457	0	-4.10931	molecular_function	Zn2Cys6 transcriptional regulator
59272	-3.1929	0	-4.67969	cellular_component	MFS permease
124084	-3.18153	0	-3.67599	#N/D	unknown protein
124296	-3.1663	0	-6.52676	#N/D	unique protein
54694	-3.14174	0	-6.8114	molecular_function	aryl-alcohol dehydrogenases
112328	-3.12313	0	-4.3494	biological_process	peptidase M14
77770	-3.09909	0	-5.15279	molecular_function	Zinc-containing alcohol dehydrogenase
103442	-3.07321	0	-3.89509	#N/D	unique protein
77703	-3.06483	0	-3.78784	molecular_function	PDR-type ABC transporters
103015	-3.05848	0	-7.2118	molecular_function	unknown protein
59900	-3.03226	0	-3.56448	#N/D	unknown protein
112125	-2.98824	0	-6.32693	molecular_function	macrophomate synthase
82662	-2.97591	0	-5.65064	#N/D	Epl1/Sm1
60422	-2.95098	0	-4.83643	#N/D	unknown protein
123149	-2.91655	0	-6.71046	#N/D	unknown protein

5818	-2.91283	0	-4.85082	cellular_component	DSBA oxidoreductase
81586	-2.90289	0	-7.33783	#N/D	unknown protein
121350	-2.88444	0	-2.38533	#N/D	unknown protein
111442	-2.84893	0	-2.76182	#N/D	Epl1/Sm1
107112	-2.84109	0	-3.46208	#N/D	unique protein
60346	-2.81418	0	-2.94553	molecular_function	Para-aminobenzoate (PABA) synthase PabaA
109998	-2.81111	0	-3.15638	molecular_function	glutathione S-transferase
79813	-2.78456	0	-2.76273	biological_process	flavoprotein monooxygenases
106223	-2.76826	0	-2.87418	#N/D	unique protein
106556	-2.75584	0	-5.7017	cellular_component	unknown protein
111082	-2.73713	0	-4.88159	molecular_function	glutathione S transferase, 2 TM
74278	-2.69238	0	-4.02703	molecular_function	Pyridine nucleotide-disulphide oxidoreductase, class-II
121294	-2.65844	0	-2.36251	#N/D	glucan endo-1,3(4)-β-D-glucosidase
76763	-2.63528	0	-2.54007	#N/D	PTH11 GPCR
26642	-2.62105	0	-2.6937	cellular_component	MFS permease
41895	-2.59998	0	-2.94475	#N/D	glutathione-S-transferase
105752	-2.56456	0	-4.53424	cellular_component	C4-dicarboxylate transporter/malic acid transport protein
21595	-2.5572	0	-5.2441	cellular_component	MFS permease
112596	-2.55556	0	-1.41047	biological_process	flavoprotein monooxygenase
64167	-2.53695	0	-2.34246	#N/D	Sexual differentiation process protein ISP4
61526	-2.53156	0	-4.92984	#N/D	unknown protein
78223	-2.52359	0	-2.0371	biological_process	Cytochrome P450 CYP3/CYP5/CYP6/CYP9 subfamilies
112665	-2.51918	0	-4.57152	molecular_function	GCN5-related N-acetyltransferase
73937	-2.51795	0	-3.13865	biological_process	Ornithine carbamoyltransferase OTC/ARG3
64330	-2.5098	0	-2.65134	molecular_function	indoleamine 2,3-dioxygenase
82616	-2.48715	0	-2.40386	biological_process	GH5 membrane bound endoglucanase CEL5b
122992	-2.48387	0	-1.5487	#N/D	GT 31 ß-glycosyltransferase
122074	-2.47668	0	-3.44861	biological_process	flavoprotein monooxygenases

105775	-2.46777	0	-3.55621	#N/D	unknown protein
31611	-2.45334	0	-3.40415	#N/D	SSCRP
23209	-2.44591	0	-1.91302	molecular_function	5'-nucleotidase
53868	-2.41506	0	-6.6063	molecular_function	NADH:flavin oxidoreductase/NADH oxidase
81362	-2.39793	0	-2.54368	molecular_function	Lipoate synthase
66657	-2.3898	0	-3.19249	cellular_component	MFS permease
3327	-2.38723	0	-1.9586	molecular_function	NADH:flavin oxidoreductase/NADH oxidase
112202	-2.36645	0	-2.83081	molecular_function	Zn2Cys6 transcriptional regulator
22426	-2.36376	0	-2.60187	molecular_function	unknown protein
121126	-2.34906	0	-2.2851	#N/D	unknown protein
120195	-2.34846	0	-2.20995	biological_process	unknown protein
52489	-2.34229	0	-1.45309	cellular_component	cytosin/purin permease
21416	-2.32478	0	-3.60418	#N/D	unknown protein
122350	-2.25454	0	-1.50573	molecular_function	Glutamate decarboxylase
59553	-2.2252	0	-2.69696	#N/D	unknown protein
110653	-2.19993	0	-3.92627	#N/D	unknown protein
122505	-2.19838	0	-2.72285	molecular_function	aryl-alcohol dehydrogenase
59014	-2.19297	0	-3.8823	molecular_function	PDR-type ABC transporters
82633	-2.17318	0	-2.07142	biological_process	GH72 β-1 3-glucanosyltransferase
76910	-2.15197	0	-2.27828	#N/D	monocarboxylate transporter
66804	-2.13527	0	-1.50367	#N/D	GH69: candidate a-glycosyltransferase
103028	-2.11697	0	-1.98288	#N/D	unique protein
123174	-2.11182	0	-2.85769	molecular_function	Phosphoglycerate dehydrogenase and related dehydrogenases; RibA GTP cyclohydrolase II
122487	-2.10717	0	-1.6775	#N/D	DNAse
105291	-2.10603	0	-5.46019	#N/D	unknown protein
3488	-2.10282	0	-2.43992	#N/D	unknown protein
56853	-2.08346	0	-1.24387	biological_process	unknown protein

60847	-2.07842	0	-2.63092	molecular_function	Mitochondrial F1F0-ATP synthase, subunit c/ATP9/proteolipid
32798	-2.07734	0	-4.45794	#N/D	unknown protein
63272	-2.07455	0	-2.43179	#N/D	NifU-like protein
70311	-2.07289	0	-1.13947	biological_process	flavoprotein monooxygenase
64685	-2.03363	0	-2.53427	#N/D	mitochondrial hypoxia responsive domain-containing protein
121820	-2.02328	0	-1.60887	molecular_function	Methionine_syntMethionine synthasevitamin-B independent
56860	-2.02039	0	-2.85848	#N/D	unknown protein
2100	-2.01609	0	-2.65602	#N/D	unknown protein DUF1531
122993	-2.01413	0	-1.71438	#N/D	GT2 polysaccharide-forming ß-glycosyltransferase; distantly animal hyaluronan synthase
80115	-2.00353	0	-2.18183	molecular_function	unknown protein
74060	-1.99083	0	-2.98363	#N/D	SSCRP
66888	-1.98822	0	-4.00234	#N/D	GT α-1,3-mannosyltransferase
72788	-1.97171	0	-4.08934	#N/D	GT 31 glycosyltransferase
81014	-1.94425	0	-1.85062	biological_process	NRPS
104968	-1.93931	0	-1.59504	#N/D	unknown protein
53495	-1.93355	0	-2.42573	#N/D	unknown protein
111832	-1.91671	0	-1.9407	molecular_function	Isoflavone reductase superfamily protein
65817	-1.89609	0	-2.95989	#N/D	GT α-1,3-mannosyltransferase CMT1
124030	-1.89053	0	-2.44335	biological_process	unknown protein with TIM barrel
123588	-1.88654	0	-2.86803	biological_process	electron transport protein, probably involved in cytochrome C assembly
121605	-1.85658	0	-1.7966	#N/D	unknown protein
5088	-1.85479	0	-3.15071	#N/D	GCN5-related acetyltransferase
23090	-1.85281	0	-1.46073	molecular_function	short chain dehydrogenase/reductase
121686	-1.84534	0	-1.83697	biological_process	Serine hydroxymethyltransferase
54554	-1.84317	0	-1.76046	biological_process	GTP cyclohydrolase 1
61763	-1.84004	0	-1.5131	#N/D	unknown protein
77138	-1.83685	0	-1.93101	#N/D	unknown protein

3641	-1.82154	0	-2.35792	biological_process	Cystathionine beta-lyases/cystathionine gamma-synthases
107936	-1.81717	0	-3.67393	cellular_component	MFS permease
81896	-1.80399	0	-1.94395	biological_process	aspartate kinase
104890	-1.80246	0	-3.07168	biological_process	4-nitrophenylphosphatase
78576	-1.79988	0	-1.52237	#N/D	unknown protein
111023	-1.79983	0	-2.25042	#N/D	unknown protein
44476	-1.79851	0	-1.96538	molecular_function	MRP-type ABC transporter
55335	-1.79648	0	-1.88544	molecular_function	nucleotide binding protein Nbp35, putative
60849	-1.79005	0	-1.70267	#N/D	FAD-dependent sulfhydryl oxidase Erv1
45369	-1.78503	0	-2.72842	#N/D	unknown protein
104513	-1.75773	0	-1.05653	molecular_function	Zn2Cys6 transcriptional regulator
82049	-1.75725	0	-2.74914	#N/D	unknown protein
77227	-1.75022	0	-2.86811	molecular_function	UDP-glucose ceramide glucosyltransferase
123475	-1.74739	0	-2.72893	#N/D	cell wall Thr-rich mannoprotein. Distantly related to S. cerevisiae Dan4p.
63397	-1.74736	0	-2.70603	molecular_function	unknown protein
105003	-1.74125	0	-1.22163	#N/D	unknown protein
102593	-1.73799	0	-1.6664	#N/D	Mitochondrial ribosomal protein L17
82516	-1.72305	0	-2.55261	biological_process	Pentafunctional Aromatic Polypeptide
105911	-1.71227	0	-1.029	#N/D	unknown protein
121706	-1.70915	0	-2.28887	biological_process	DAHP synthase ARO4
60086	-1.70567	0	-3.31215	cellular_component	MFS permease
68178	-1.6994	0	-1.95953	molecular_function	unknown protein, HAD superfamily hydrolase
58264	-1.69916	0	-2.44035	#N/D	paxU orthologue ? (indole-terpene biosynthesis?)
46238	-1.68011	0	-1.29244	biological_process	S-adenosylmethionine synthetase
82087	-1.67595	0	-1.56641	#N/D	unknown protein
53567	-1.67402	0	-2.08358	molecular_function	glutathione reductase
104182	-1.67279	0	-2.47742	molecular_function	Zn2Cys6 transcriptional regulator
105070	-1.67142	0	-1.96799	biological_process	MetC Cystathionine beta-lyases/cystathionine gamma-synthases

22885	-1.66575	0	-2.10092	biological_process	ATP11 protein
59598	-1.6642	0	-1.41297	#N/D	unknown protein
39827	-1.66358	0	-1.94245	#N/D	unknown protein
49205	-1.63287	0	-1.79063	biological_process	cytochrome C peroxidase
1993	-1.61952	0	-2.27962	biological_process	γ-glutamyl phosphate reductase GPR
66844	-1.61572	0	-1.41489	molecular_function	GPI ethanolamine phosphate transferase, putative
109435	-1.61121	0	-1.355	molecular_function	unknown protein, 1 TM
81019	-1.61053	0	-3.27378	molecular_function	Zinc-containing alcohol dehydrogenase
122646	-1.57966	0	-1.46319	molecular_function	Alpha/beta hydrolase
123999	-1.57832	0	-1.4389	molecular_function	NADH:flavin oxidoreductase/NADH oxidase
106081	-1.57578	0	-2.14781	biological_process	unknown protein
74026	-1.57296	0	-1.88058	biological_process	CDP-alcohol phosphatidyltransferase
102607	-1.5718	0	-2.22927	#N/D	unknown protein
65559	-1.566	0	-1.16554	molecular_function	Respiratory-chain NADH dehydrogenase, 51 kDa subunit
102863	-1.56471	0	-1.78455	#N/D	unknown protein
56176	-1.56466	0	-1.54699	molecular_function	MRP-type ABC transporter
74892	-1.55834	0	-1.1602	#N/D	ATP synthase regulation protein NCA2, putative
72685	-1.55125	0	-1.95449	biological_process	6-phosphogluconate dehydrogenase, decarboxylating
110620	-1.54346	0	-1.45583	biological_process	unknown protein
23420	-1.53161	0	-1.4672	molecular_function	prohibitin PHB1
122857	-1.5219	0	-1.20483	#N/D	prohibitin-2
106150	-1.52161	0	-1.33583	#N/D	Coenzyme Q (ubiquinone) biosynthesis protein Coq4, putative
77629	-1.51498	0	-1.41151	#N/D	unknown protein
53318	-1.51237	0	-1.53434	biological_process	cytochrome c oxidase subunit Va
121534	-1.50521	0	-1.38958	molecular_function	pyruvate decarboxylase
104609	-1.50498	0	-1.2099	molecular_function	UbiE/COQ5 ubiquinone methyltransferase
119819	-1.5047	0	-1.98762	cellular_component	GCPR, mPR-type
110655	-1.50152	0	-1.76492	#N/D	RhoA GTPase effector arrestin

107667	-1.49853	0	-2.04624	#N/D	unknown protein
109929	-1.49703	0	-2.23523	#N/D	unknown protein
80932	-1.49393	0	-2.0137	molecular_function	Cytochrome c oxidase assembly protein CtaG/Cox11
119642	-1.49302	0	-1.30225	#N/D	unknown protein
75769	-1.49235	0	-1.82329	biological_process	glucose-6-phosphate dehydrogenase
46240	-1.48822	0	-1.69227	molecular_function	ATP-binding protein
51430	-1.48582	0	-1.65978	biological_process	ribosomal protein L10e.
119735	-1.4789	0	-1.48177	molecular_function	Glyceraldehyde-3-phosphate dehydrogenase, isozyme 2
103798	-1.47555	0	-2.15466	#N/D	SSCRP
80312	-1.4737	0	-2.38614	#N/D	unknown protein
71343	-1.47124	0	-1.163	biological_process	cytochrome oxidase c subunit VIb
75334	-1.46871	0	-1.10779	#N/D	unknown protein
78970	-1.46619	0	-2.44629	cellular_component	mitochondrial (phosphate) carrier
50947	-1.46235	0	-2.02743	#N/D	cell division cycle protein 123 , putative
67859	-1.46082	0	-1.60516	biological_process	aspartyl-tRNA synthase.
65646	-1.45949	0	-2.02194	#N/D	GT α-1,6-mannosyltransferase
76551	-1.4501	0	-1.46704	molecular_function	uridylate kinase
67806	-1.4471	0	-2.04752	cellular_component	Amino acid permease
63742	-1.44652	0	-1.49252	biological_process	Demethoxyubiquinone hydroxylase
4668	-1.44454	0	-2.17858	cellular_component	cytochrome c oxidase assembly protein
4654	-1.44176	0	-2.24487	molecular_function	Histidinol dehydrogenase
106147	-1.43954	0	-1.5642	#N/D	unknown protein
121664	-1.43726	0	-1.8269	molecular_function	Glutamate decarboxylase and related proteins
111516	-1.43328	0	-1.49176	#N/D	unknown protein
78561	-1.433	0	-1.18214	biological_process	Molybdenum cofactor biosynthesis protein
57357	-1.43075	0	-1.74163	biological_process	Glycine cleavage T protein (aminomethyl transferase), putative
104704	-1.43072	0	-1.26684	biological_process	60S ribosomal protein RML2, mitochondrial precursor from Ashbya gossypii.
47136	-1.42927	0	-1.88052	biological_process	Glutathione peroxidase

28050	-1.42914	0	-1.80546	biological_process	unknown protein
22632	-1.42184	0	-1.64201	molecular_function	aconitate hydratase
111538	-1.41962	0	-1.30424	#N/D	unknown protein
73783	-1.41578	0	-1.64168	molecular_function	mitochondrial co-chaperone GrpE , putative
122501	-1.41513	0	-1.89173	#N/D	unknown protein
81188	-1.41482	0	-1.5369	molecular_function	HisF Imidazoleglycerol-phosphate synthase
79334	-1.4137	0	-1.86638	biological_process	NADH-cytochrome b5 reductase, putative
76775	-1.41135	0	-1.25161	cellular_component	MFS permease
119759	-1.4084	0	-1.59938	biological_process	BZIP transcriptional regulator
4981	-1.40181	0	-2.15834	biological_process	dihydrounknown protein-acid dehydratase
121890	-1.39697	0	-1.62199	biological_process	mitochondrial processing peptidase, α-subunit
111351	-1.3915	0	-2.27613	biological_process	DHBP_synthase,-dihydroxy--butanone -phosphate synthase
29439	-1.39048	0	-2.30924	#N/D	unknown protein
58612	-1.38696	0	-2.24693	biological_process	ounknown proteinsterol binding protein
120911	-1.3851	0	-2.24452	molecular_function	short chain dehydrogenase/reductase
119609	-1.38437	0	-2.12865	biological_process	Phospho-2-dehydro-3-deoxyheptonate aldolase
119788	-1.38274	0	-2.24353	molecular_function	saccharopine dehydrogenase
123729	-1.38204	0	-1.79667	molecular_function	malate dehydrogenase
122043	-1.38017	0	-2.21374	molecular_function	unknown protein
122641	-1.37691	0	-2.257	biological_process	nicotinate phosphoribosyltransferase
46301	-1.373	0	-1.02363	biological_process	ubiquinone biosynthesis monooxygenase COQ6
60406	-1.36744	0	-2.14216	biological_process	mitochondrial matrix iron-sulfur protein
79037	-1.36487	0	-2.0593	biological_process	asparagine synthase
59023	-1.36362	0	-1.63749	molecular_function	Fe superoxide dismutase
119974	-1.36189	0	-1.39229	molecular_function	zinc-binding dehydrogenase, putative
82434	-1.36084	0	-1.60428	#N/D	Unknown protein with TPR structural motif.
21609	-1.35902	0	-1.30241	cellular_component	Ubiquinol cytochrome reductase
21270	-1.35842	0	-1.41607	#N/D	CAP20 virulence factor

57430	-1.35729	0	-1.40963	biological_process	Glutamate-cysteine ligase
78679	-1.35427	0	-2.29916	cellular_component	mitochondrial carrier protein
55055	-1.35423	0	-1.10859	biological_process	MTHFRMethylenetetrahydrofolate reductase
120556	-1.35325	0	-1.41	cellular_component	Mitochondrial substrate carrier
107551	-1.35208	0	-1.81106	molecular_function	unknown protein
39755	-1.3516	0	-2.0673	biological_process	GH16 glucan endo-1,3(4)-β-D-glucosidase
80086	-1.34361	0	-1.18933	cellular_component	MFS peptide transporter
74346	-1.34145	0	-1.80981	biological_process	Translation elongation factor precursor from Aspergillus fumigatus.
44386	-1.33896	0	-1.34505	#N/D	ribosomal protein MRPL40.
78956	-1.33556	0	-1.26621	#N/D	unknown protein
4901	-1.33191	0	-1.57045	#N/D	cytochrome c oxidase assembly protein COX16, putative
81690	-1.33182	0	-1.23271	#N/D	ß-arrestin protein, shares similarity with Aspergillus idulans CreD, possible inhibitor of G-protein coupled receptors
78049	-1.33143	0	-1.342	molecular_function	elongation factor Tu (G).
63398	-1.33143	0	-1.20684	cellular_component	Peptidase M48, Ste24p
120968	-1.32781	0	-2.0504	molecular_function	Copper chaperone for superoxide dismutase
22251	-1.32389	0	-2.23711	cellular_component	Mitochondrial carnitine-acylcarnitine carrier protein
75294	-1.32084	0	-1.89197	biological_process	BCAT_beta_family
77284	-1.31408	0	-2.12082	molecular_function	GH12 endo-ß-1,4-glucanase
59011	-1.31107	0	-1.29654	biological_process	#N/D
74782	-1.31065	0	-1.73039	#N/D	survival factor 1
122569	-1.30724	0	-1.31205	#N/D	unknown protein
75116	-1.30628	0	-2.23409	cellular_component	Sur4p fatty acid elongase ofSaccharomyces cerevisiae
65333	-1.30564	0	-1.38223	biological_process	GH15 alpha-glycosidase (Glucoamylase and related glycosyl hydrolases)
70397	-1.30102	0	-1.34178	biological_process	alanyl-transfer RNA synthetase.
58077	-1.29978	0	-1.5119	#N/D	unknown protein
119898	-1.29482	0	-1.75914	molecular_function	ribosome biogenesis GTPase Lsg1
52924	-1.28842	0	-2.14285	biological_process	transcriptional activator, zinc finger, NF-X1-type

53981	-1.28337	0	-1.7464	#N/D	unknown protein
63269	-1.28122	0	-1.02066	biological_process	mitochondrial ribosomal protein MRPL3.
57947	-1.28052	0	-1.82284	molecular_function	BioF -keto--aminopelargonate synthetase and related enzymes
120730	-1.27989	0	-1.11274	biological_process	unknown protein
54437	-1.27979	0	-2.05064	molecular_function	Zn2Cys6 transcriptional regulator
124097	-1.27767	0	-1.26212	biological_process	phenazine biosynthesis protein phzF
121345	-1.27661	0	-1.70277	molecular_function	SerC Phosphoserine aminotransferase
78347	-1.27513	0	-1.55539	biological_process	3-deoxy-7-phosphoheptulonate synthase
111074	-1.27478	0	-1.24989	molecular_function	GAL7 UDP glucose-1-phosphate galactosyltransferase
79972	-1.27422	0	-1.61257	#N/D	unknown protein
82360	-1.27065	0	-1.01856	molecular_function	lipase/serine esterase, putative
77476	-1.27063	0	-1.16948	#N/D	unknown protein
75200	-1.26777	0	-1.55846	#N/D	unknown protein
69290	-1.26682	0	-1.5288	biological_process	Asparaginase, Asparaginase (amidohydrolase); 3' domain cd00204, ANK, ankyrin repeats
62213	-1.26211	0	-1.69093	biological_process	unknown protein with fasciclin domain
23431	-1.25459	0	-1.41822	molecular_function	Cytochrome bd ubiquinol oxidase, 14 kDa subunit
45675	-1.25118	0	-1.33419	molecular_function	unknown protein
58651	-1.24936	0	-1.57173	biological_process	adenylosuccinate synthase
32473	-1.24925	0	-1.2873	#N/D	mitochondrial ribosomal protein RSM25.
77656	-1.24761	0	-1.18815	biological_process	phosphoglycerate mutase
33273	-1.24526	0	-1.15326	biological_process	#N/D
5436	-1.24522	0	-1.39072	#N/D	unknown protein containing major histocompatibility complex
60793	-1.24429	0	-1.37992	biological_process	unknown protein
60814	-1.24126	0	-1.0285	molecular_function	glutamyl-tRNA amidotransferase subunit B in other fungi.
74449	-1.24117	0	-1.14231	biological_process	unknown protein
120712	-1.23516	0	-1.06373	biological_process	3-oxoacyl-(acyl carrier protein) synthase
108459	-1.23154	0	-1.75374	molecular_function	carbamoyl-phosphate synthase (glutamine-hydrolyzing)arginine-specific large chain

78661	-1.22929	0	-1.08903	biological_process	ribosomal protein S5, MRPS5.
69222	-1.22679	0	-1.3645	#N/D	unknown protein
81097	-1.22631	0	-2.14568	cellular_component	unknown protein with TLC domain
67300	-1.22071	0	-1.30779	molecular_function	unknown protein
69863	-1.21968	0	-2.12663	molecular_function	Amidase
120635	-1.21916	0	-1.27064	molecular_function	transketolase-like protein
74020	-1.21493	0	-1.22686	biological_process	Orotidine 5'-phosphate decarboxylase
22830	-1.21386	0	-2.52165	molecular_function	Glutathione S-transferase
66047	-1.21093	0	-1.72354	biological_process	Zn2Cys6 transcriptional regulator
103132	-1.2093	0	-1.95924	molecular_function	acetylglutamate kinase ARG6
69179	-1.20876	0	-1.2427	#N/D	molecular chaperone, contains ABC-1 domain
5233	-1.20648	0	-1.59782	molecular_function	Aspartate/otherAminotransferase
56559	-1.20017	0	-1.0837	#N/D	unknown protein
58493	-1.19967	0	-1.23024	biological_process	Cytochrome c oxidase, subunit Vb
123805	-1.19954	0	-1.79482	biological_process	DHBP_synthase,-dihydroxy--butanone -phosphate synthase
76288	-1.19813	0	-1.76281	molecular_function	short chain dehydrogenase/reductase
5917	-1.19585	0	-1.31848	#N/D	unknown protein
54633	-1.19337	0	-1.54549	#N/D	GT ß-glycosyltransferases
69164	-1.19191	0	-1.10718	#N/D	MFS permease
82385	-1.19184	0	-1.17367	biological_process	methionyl-tRNA synthetase.
79565	-1.18837	0	-1.81619	molecular_function	Thioredoxin reductase TrxB
59579	-1.18673	0	-1.60407	#N/D	unknown protein
77955	-1.1864	0	-1.07021	#N/D	cytochrome b5-like Heme/Steroid binding domain-containing protein
59919	-1.1862	0	-1.34027	#N/D	RNAse III domain protein
121248	-1.18074	0	-1.43121	molecular_function	ATP12 chaperone , putative
109601	-1.1789	0	-1.04628	#N/D	unknown protein
23184	-1.17661	0	-2.04903	molecular_function	Isocitrate/isopropylmalate dehydrogenase
3129	-1.17582	0	-1.2232	#N/D	alpha-tubulin suppressor protein Aats1

74486	-1.17413	0	-1.59568	#N/D	unknown protein
42063	-1.17094	0	-2.22529	#N/D	unknown protein
28159	-1.17002	0	-1.49585	cellular_component	Protoheme IX farnesyltransferase
50429	-1.16865	0	-1.61097	#N/D	unknown protein
65411	-1.16817	0	-1.21856	biological_process	nicotinate-nucleotide diphosphorylase
50996	-1.16773	0	-1.86509	#N/D	unknown protein, C2 domain
58815	-1.16723	0	-1.49034	molecular_function	alternative NADH-dehydrogenase, putative
2365	-1.1667	0	-1.33655	#N/D	Unknown protein
77188	-1.16301	0	-1.19391	biological_process	60S ribosomal protein L1
30805	-1.16107	0	-1.22907	molecular_function	endonuclease III-like excision repair N-glycosylase involved in the repair of DNA base damage
77242	-1.15889	0	-1.8077	biological_process	glutamate N-acetyltransferase precursor
120143	-1.15433	0	-1.54315	molecular_function	chaperone DnaJ
60850	-1.15081	0	-1.18868	biological_process	Mitochondrial initiation factor 2 (IF-2).
53091	-1.14915	0	-1.14334	biological_process	Threonine/serine dehydratases
119695	-1.14497	0	-1.10338	molecular_function	RNA:NAD 2'-phosphotransferase TPT1.
55752	-1.14408	0	-1.08515	biological_process	ribosomal protein L29. Shows amino acid sequence similarity to S. cerevisiae mitochondrial ribosomal protein MRPL4.
66163	-1.13921	0	-1.0725	#N/D	unknown protein
63419	-1.13729	0	-1.06745	#N/D	Met-10+ like-protein
75935	-1.13164	0	-1.64859	#N/D	unknown protein
40290	-1.13124	0	-1.1192	#N/D	unknown protein
120332	-1.12842	0	-1.08027	#N/D	unknown protein
46446	-1.12205	0	-1.13152	#N/D	Thioredoxin binding protein TBP-2
122296	-1.11875	0	-2.0791	molecular_function	citrate (Si)-synthase
52156	-1.11806	0	-1.55717	#N/D	unknown protein
73523	-1.1178	0	-1.01313	biological_process	unknown protein with WSC domains
64818	-1.11515	0	-1.88038	cellular_component	Mitochondrial substrate carrier

22799	-1.10999	0	-1.14693	molecular_function	unknown protein
70439	-1.10614	0	-1.01658	molecular_function	NADH-ubiquinone oxidoreductase
120705	-1.10299	0	-1.61868	#N/D	unknown protein
47829	-1.10047	0	-1.68142	cellular_component	unknown protein
75226	-1.10028	0	-1.1287	biological_process	dihydroorotate dehydrogenase
112556	-1.09888	0	-1.51911	#N/D	UTP5, encoding a component of the SSU processome
62470	-1.09653	0	-1.57428	molecular_function	mitochondrial elongation factor G.
122210	-1.09038	0	-1.36534	biological_process	ribosomal protein S4.
111536	-1.08952	0	-1.45638	biological_process	unknown protein
22013	-1.08924	0	-1.52899	molecular_function	Hsp70 chaperone (BiP), putative
2648	-1.08807	0	-1.15449	biological_process	Fe(II)/2-oxoglutarate-dependent diounknown proteingenase
63709	-1.08575	0	-1.71802	#N/D	cystathionine beta-synthase (beta-thionase), putative
55252	-1.08322	0	-1.11083	molecular_function	unknown protein
80854	-1.0817	0	-1.18463	#N/D	ribosomal protein MRPL24.
68728	-1.07334	0	-1.10538	biological_process	hexaprenyl pyrophosphate synthase
80922	-1.07309	0	-1.34912	molecular_function	unknown protein
73571	-1.07173	0	-1.17983	biological_process	Cytochrome c1, heme protein, mitochondrial
70040	-1.06989	0	-1.00783	biological_process	Bet4p, alpha subunit of geranygeranyltransferase required for vesicle traffic between ER and Golgi
102744	-1.06971	0	-1.78604	molecular_function	TrpE Anthranilate/para-aminobenzoate synthases component I
3394	-1.06923	0	-2.10451	molecular_function	unknown protein
108893	-1.06385	0	-2.11945	cellular_component	MFS permease
78585	-1.06264	0	-5.1605	cellular_component	MFS permease
57141	-1.06056	0	-1.02217	biological_process	FAD binding domain-containing protein
81473	-1.0595	0	-1.42433	#N/D	caffeine-induced death protein 2
120064	-1.0591	0	-1.59236	molecular_function	DEAD box helicase Hel1
71759	-1.05886	0	-1.1933	biological_process	tyrosyl-tRNA synthetase, class Ib.
22841	-1.0581	0	-1.21254	#N/D	unknown protein

122226	-1.05779	0	-1.77051	molecular_function	Carbamoyl-phosphate synthase, small chain
22464	-1.0568	0	-1.49927	biological_process	Bifunctional P-450:NADPH-P450 reductase
41428	-1.05393	0	-1.5512	biological_process	Tyrosine specific protein phosphatase and dual specificity protein phosphatase
67616	-1.05328	0	-1.64063	#N/D	unknown protein
104334	-1.05281	0	-1.23251	#N/D	unknown protein
120568	-1.04992	0	-1.35613	biological_process	enolase
102382	-1.04549	0	-1.46191	molecular_function	hydroxyacylglutathione hydrolase
105589	-1.04369	0	-1.1783	biological_process	mitochondrial ATPase inhibitor, putative
60008	-1.03483	0	-1.22481	#N/D	ribosomal protein MRPL35.
120248	-1.0345	0	-1.01622	#N/D	unknown protein
72918	-1.02632	0	-1.10111	biological_process	ribosomal protein L19
109234	-1.02578	0	-1.10142	#N/D	D-aminopeptidase
60698	-1.02535	0	-2.10391	#N/D	unknown protein
111102	-1.02405	0	-1.87731	biological_process	Phospholipase C, related to Listeria monocytogenesPlcA (Evalue 1.62e-21), has a homologue in Gibberella zeae
123758	-1.02286	0	-2.01987	molecular_function	unknown protein
48883	-1.02098	0	-1.30316	biological_process	unknown protein
79359	-1.02048	0	-1.32979	#N/D	unknown protein
68636	-1.01956	0	-1.56071	biological_process	ribosomal protein S11
74158	-1.00682	0	-1.70886	molecular_function	ribosome biogenesis protein Ssf2, putative
81906	-1.00328	0	-1.1258	molecular_function	unknown protein
1751	-1.00272	0	-1.62109	biological_process	FAD monooxygenase
120272	-1.00141	0	-1.64685	molecular_function	64 kDa mitochondrial NADH dehydrogenase, putative

Table S3.3. Genes from sophorose regulon

protein id	qmCelGlu	qmSphCel	qmSphGlu	GOTerm	Description
106164	0	2.026102	10.00704	molecular_function	short chain dehydrogenase/reductase
59628	0	3.845626	5.711052	#N/D	unknown protein
48444	0	2.108511	5.524488	cellular_component	MFS maltose permease
5345	0	2.578698	5.44315	molecular_function	FAD-containing oxidoreductase
122087	0	2.897986	5.225164	cellular_component	unknown protein
22915	0	1.156308	4.544159	molecular_function	glucose oxidase
60945	0	1.340636	4.450755	cellular_component	MFS permease
55802	0	1.955924	4.440557	molecular_function	GH76 α-1,6-mannanase
67971	0	2.759523	4.248074	#N/D	MYND-type Zn-finger protein
69026	0	1.068203	4.044023	cellular_component	MFS permease
34985	0	2.215772	3.863322	biological_process	C-5 cytosine-specific DNA methylase
21876	0	5.034468	3.827983	molecular_function	Zinc-binding oxidoreductase
107947	0	1.687466	3.72002	biological_process	Acyl-CoA synthetase
59801	0	3.167657	3.569349	#N/D	unknown protein
62576	0	2.153018	3.562405	biological_process	alpha/beta hydrolase
80028	0	1.972476	3.533757	molecular_function	MRP-type ABC transporter
80003	0	2.077766	3.471495	molecular_function	Phosphoglycerate dehydrogenase and related dehydrogenases
76359	0	4.406915	3.400422	#N/D	unknown protein, only present in ascomycota
62165	0	3.54018	3.375828	molecular_function	catechol dioxigenase
110220	0	2.426367	3.340595	molecular_function	unknown protein
4146	0	3.41781	3.323454	biological_process	flavoprotein monooxygenase
109317	0	1.478546	3.26139	#N/D	unknown protein
123881	0	2.24301	3.209907	molecular_function	Zn2Cys6 transcriptional regulator

120851	0	2.350154	3.209781	#N/D	HET domain protein, related to N. crassa pin-c3
78462	0	2.474063	3.149371	biological_process	L-carnitine dehydratase/alpha-methylacyl-CoA racemase
69611	0	1.511538	3.123586	cellular_component	MFS permease
31134	0	1.728448	3.055132	cellular_component	isoprenylcysteine carboxyl methyltransferase
64710	0	3.313531	3.041638	molecular_function	AAA+-type ATPase
53776	0	2.06092	3.028389	biological_process	Protein kinase
56830	0	1.690465	2.990558	molecular_function	dipeptidyl peptidase 5
28409	0	2.547462	2.988303	cellular_component	MFS permease
108583	0	2.179258	2.987557	#N/D	unknown protein
72321	0	2.572404	2.928106	biological_process	subtilisin-like serine protease
59665	0	1.011742	2.898376	#N/D	unknown protein
123865	0	2.790228	2.898022	biological_process	Peptidase S8 and S53, subtilisin, kexin, sedolisin
106171	0	2.244674	2.893039	#N/D	HET protein
82619	0	1.473093	2.881628	biological_process	arginosuccinate synthetase
123009	0	2.240763	2.88061	#N/D	glutamine synthetase
111758	0	2.550583	2.770942	#N/D	unknown protein
55274	0	1.47845	2.754819	molecular_function	Zn2Cys6 transcriptional regulator
58584	0	1.35696	2.707003	cellular_component	aquaglyceroporin
121839	0	2.378594	2.695666	molecular_function	HSP70
81082	0	2.339623	2.672165	cellular_component	aquaglyceroporin
105956	0	2.128636	2.650193	molecular_function	GH13 α-amylase
61212	0	1.03104	2.630433	molecular_function	cytochrome P450 monooxygenase
58772	0	1.325385	2.616081	biological_process	cytochrome P450 monooxygenase
52206	0	1.23742	2.607064	#N/D	#N/D
60981	0	2.530751	2.606499	molecular_function	unknown protein
121843	0	2.192333	2.555543	#N/D	C6HC zinc finger protein
122792	0	4.270018	2.537278	#N/D	unknown protein
102998	0	1.968667	2.512345	molecular_function	ADP-ribosylglycohydrolase-like protein

120031	0	1.01981	2.430003	#N/D	unknown protein
43129	0	1.617138	2.418937	#N/D	unknown protein
77254	0	1.995884	2.404978	#N/D	unknown protein
70630	0	1.186958	2.375677	#N/D	homoserine acetyltransferase family protein
77288	0	1.767526	2.374451	molecular_function	Thioredoxin reductase
77749	0	1.368702	2.335881	biological_process	fumarylacetoacetase
109337	0	2.212085	2.328454	#N/D	unknown protein
124222	0	1.615815	2.317764	molecular_function	CaaX-protease, related to E. nidulans rce1, involved in signal transduction
120311	0	1.778977	2.309092	#N/D	unique protein
47286	0	1.379713	2.267705	biological_process	unknown protein
81511	0	2.987116	2.265545	molecular_function	formamidase
123673	0	3.345551	2.265287	biological_process	Ankyrin
111202	0	1.488771	2.260612	#N/D	unknown protein
65891	0	1.586424	2.259524	biological_process	PKS
59778	0	1.486868	2.237939	#N/D	GPCR, related to A nidulans GprC
120473	0	1.338093	2.184033	molecular_function	Dihydrolipoamide transacylase (alpha-keto acid dehydrogenase E2 subunit)
76690	0	2.870982	2.155443	#N/D	unknown protein
123797	0	1.538318	2.155234	#N/D	unique protein
103822	0	2.48041	2.151354	#N/D	unknown protein
123978	0	1.023667	2.14463	molecular_function	GMC methanol oxidase
120381	0	1.583068	2.13777	#N/D	unique protein
122147	0	2.591042	2.11589	#N/D	unique protein
123261	0	2.289259	2.103846	#N/D	unknown protein
62693	0	1.326319	2.092948	molecular_function	ABC-transporter Ste6p
56215	0	1.807432	2.078175	#N/D	26S proteasome non-ATPase regulatory subunit 9, putative
122941	0	1.689835	2.005356	#N/D	unique secreted protein with CFEM domain
110452	0	2.072907	1.963019	#N/D	unknown protein
73654	0	1.253233	1.962791	biological_process	BZIP transcriptional regulator

77785	0	1.02121	1.946694	cellular_component	MFS permease
65380	0	1.001023	1.944784	cellular_component	GH47 α-1,2-mannosidase
103149	0	1.79412	1.942495	molecular_function	AAA ATPase
5446	0	1.522403	1.935772	#N/D	unknown protein
66280	0	1.536101	1.929641	molecular_function	unknown protein
61517	0	1.694858	1.927565	#N/D	unknown protein
120821	0	2.088298	1.924949	molecular_function	phytase
78463	0	1.493554	1.914814	#N/D	unknown protein
62244	0	1.898438	1.909129	molecular_function	Zn2Cys6 transcriptional regulator
80231	0	1.569983	1.90621	biological_process	glucokinase
22590	0	2.973734	1.897229	#N/D	unknown protein
121107	0	1.680571	1.88105	molecular_function	Zn2Cys6 transcriptional regulator
106626	0	2.61631	1.876801	molecular_function	unknown protein, SWIFT domain
109078	0	1.081755	1.857685	molecular_function	prenyltransferase, related to E. nidulans CaaX farnesyltransferase beta subunit ram1
109945	0	3.110548	1.856267	#N/D	unique protein
47603	0	1.098248	1.8424	molecular_function	succinyl-CoA:3-ketoacid-coenzyme A transferase subunit A
122124	0	2.799162	1.832835	molecular_function	AAA ATPase
55041	0	1.215527	1.820423	#N/D	unknown protein
56726	0	1.355274	1.769996	molecular_function	Branched chain alpha-keto acid dehydrogenase complex, alpha subunit
22000	0	1.110273	1.75079	#N/D	unknown protein
80159	0	1.066614	1.748956	molecular_function	DNA-directed DNA polymerase B
104180	0	1.749748	1.747284	molecular_function	Ankyrin
50616	0	2.63594	1.743611	cellular_component	Coenzyme A transferase
105251	0	2.089133	1.733125	#N/D	unknown protein
81756	0	1.792493	1.731554	#N/D	unknown protein
65921	0	1.495932	1.730249	biological_process	Acetyl/propionyl-CoA carboxylase alpha subunit
26994	0	1.171033	1.716005	#N/D	unknown protein, with F-box/WD domains
81649	0	1.585313	1.704179	biological_process	Flavin-containing monooxygenase

64345	0	1.212592	1.700261	molecular_function	3-Methylcrotonyl-CoA carboxylase, non-biotin containing subunit/Acetyl-CoA carboxylase carboxyl transferase, subunit beta
108914	0	1.502326	1.698873	molecular_function	methyltransferase type 11
75450	0	1.591553	1.692777	molecular_function	unknown protein
120193	0	1.540725	1.675288	#N/D	unknown protein
79111	0	1.499783	1.675152	biological_process	unknown protein
119864	0	1.77281	1.667609	#N/D	unknown protein
106885	0	1.474782	1.623891	molecular_function	acyl-CoA dehydrogenase
119568	0	1.026905	1.618938	#N/D	unknown protein
65695	0	1.875665	1.592752	#N/D	unknown protein
21664	0	1.060139	1.558856	#N/D	unknown protein
112180	0	1.698802	1.50847	biological_process	unknown protein
81328	0	1.316457	1.50551	#N/D	unknown protein, only in fungi
122069	0	2.380456	1.505502	molecular_function	SSCRP
119860	0	1.560283	1.504073	#N/D	unknown protein
79671	0	1.382125	1.495402	molecular_function	N-acetyl-glucosamine-6-phosphate deacetylase
58213	0	1.2988	1.489838	biological_process	Ku80p (Hdf2p)
112536	0	1.572653	1.476576	#N/D	unknown protein
61995	0	1.189866	1.458073	#N/D	unknown protein
36608	0	1.885512	1.450693	#N/D	unknown protein
122745	0	1.084886	1.450507	molecular_function	2-oxoisovalerate dehydrogenase subunit beta, putative
50583	0	1.114966	1.450184	biological_process	Guanine deaminase (GDEase), an aminohydrolase responsible for the conversion ofGuanine to xanthine and ammonia, the first step to utilizeGuanine as a nitrogen source.
123262	0	1.557088	1.441768	molecular_function	unique protein, HTG, amidase domain, 1 TM
21997	0	1.296896	1.435195	molecular_function	Zn2Cys6 transcriptional regulator
120057	0	1.191501	1.395717	#N/D	unknown protein
64066	0	1.02244	1.365294	molecular_function	acyltransferase 3

60033	0	1.630451	1.34066	molecular_function	short chain dehydrogenase/reductase
122146	0	1.146799	1.339181	#N/D	unknown protein
79396	0	1.870718	1.300573	#N/D	GT2 ß-glycosyltransferase, related to hyaluronan synthases
110267	0	4.486189	1.286542	biological_process	unknown protein
22115	0	1.794445	1.279311	biological_process	poly polymerase (Poly[ADP-ribose] synthetase)
111932	0	1.789072	1.229731	molecular_function	PutA delta-1-pyrroline-5-carboxylate dehydrogenase
79669	0	1.047997	1.225824	biological_process	GH3 β-N-acetylglucosaminidase
76620	0	1.827365	1.192208	cellular_component	Glycerol-3-phosphate dehydrogenase
119694	0	1.478329	1.185661	#N/D	unknown protein
64372	0	1.027651	1.176238	#N/D	unknown protein
123648	0	1.188312	1.163056	#N/D	unknown protein
68618	0	1.312159	1.158619	#N/D	unknown protein
123658	0	1.134634	1.15024	molecular_function	unique protein
2721	0	1.091888	1.141701	biological_process	unknown protein
63240	0	1.032476	1.122377	#N/D	HET-E-1, putative
66111	0	1.908369	1.116006	molecular_function	MRP-type ABC transporter
73792	0	1.039341	1.088295	molecular_function	Zn2Cys6 transcriptional regulator
22093	0	1.020072	1.073492	biological_process	Protein farnesyltransferase, alpha subunit
4231	0	2.037077	1.055927	biological_process	GATA type transcriptional regulator
81652	0	1.853322	1.038344	biological_process	unknown protein
123508	0	1.187498	1.037093	#N/D	unknown protein
124001	0	1.355082	1.000334	molecular_function	Protein phosphatase 2C/pyruvate dehydrogenase (lipoamide) phosphatase
