# Supplementary material for: Comparative metabolism of cellulose, sophorose and glucose in Trichoderma reesei using high-throughput genomic and proteomic analyses
Source: Biotechnol Biofuels. 2014 Mar 21;7:41. doi: 10.1186/1754-6834-7-41 (PMC3998047; doi:10.1186/1754-6834-7-41)
Supplement: Additional file 4: Table S4A — Carbohydrate active enzyme (CAZy) genes that are upregulated in sophorose. Table S4B. CAZy enzymes that are upregulated in celulose. Table S4C. CAZy enzymes that are upregulated in glucose. [file 1754-6834-7-41-S4.pdf]

Table S4A. CAZy genes that are up regulated in sophorose

Protein ID	Name	Class	Family	Annotation	log_2 FC Sph/Cel	p-value
105956		Glycoside hydrolase	13	Candidate α-amylase	2.1286	6E-24
55802		Glycoside hydrolase	76	Candidate α-1,6-mannanase	1.9559	8.7E-15
82235		Glycoside hydrolase	31	Candidate α-glucosidase	1.8953	8.51E-20
82227	cel3c	Glycoside hydrolase	3	Candidate β-glucosidase	1.6899	2.57E-32
120749	bgl2/cel1a	Glycoside hydrolase	1	β-glucosidase	1.6795	1.01E-18
50215		Glycoside hydrolase	16	Candidate endo-1,3-β-D-glucosidase/1,3-glucan binding protein	1.6128	1.15E-23
59082	chi18-2	Glycoside hydrolase	18	Candidate chitinase	1.5828	0.003875319
80240	bga1	Glycoside hydrolase	35	β-galactosidase	1.5736	5.07E-31
66041	chi18-18	Glycoside hydrolase	18	Candidate chitinase	1.5377	7.42E-11
106575		Glycoside hydrolase	79	Candidate β-glucuronidase	1.5174	1.22E-14
123456		Glycoside hydrolase	65	Candidate α,α-trehalase	1.5006	5.69E-17
79671		Carbohydrate esterase	9	Candidate N-acetyl-glucosamine-6-phosphate deacetylase	1.3821	0.00000921
108477		Glycoside hydrolase	13	Candidate α-glucosidase/oligo α-glucosidase	1.3297	9.49E-14
74198		Glycoside hydrolase	92	Candidate α-1,2-mannosidase	1.2728	5.09E-08
122511		Glycoside hydrolase	16	Candidate glucan endo-1,3(4)-β-D-glucosidase	1.2565	2.07E-11
121735	cel3b	Glycoside hydrolase	3	Candidate β-glucosidase	1.2107	9.89E-12
71532		Glycoside hydrolase	71	Candidate α-1 3-glucanase	1.1734	8.21E-08
22197	cel1b	Glycoside hydrolase	1	Candidate β-glucosidase	1.1577	2.97E-19
79669		Glycoside hydrolase	3	Candidate β-N-acetylglucosaminidase	1.0480	9.44E-16
58117		Glycoside hydrolase	89	Candidate α-N-acetylglucosaminidase	1.0084	0.000390284
65380		Glycoside hydrolase	47	Candidate α-1,2-mannosidase	1.0010	1.66E-13

Table S4B. CAZy enzymes that are up regulated in cellulose

Protein ID	Name	Class	Family	Annotation	log_2 FC Sph/Cel	p-value
80833	chi46	Glycoside hydrolase	18	Chitinase	-1.0173	3.2E-09
73632	axe1	Carbohydrate esterase	5	Acetyl xylan esterase	-1.0245	2.32E-08
53731		Glycoside hydrolase	5	Candidate endo-β-1,4-glucanase	-1.0746	0.006503566
65162	Endo T	Glycoside hydrolase	18	Endo-N-acetyl-β-D-glucosaminidase	-1.0917	1.46E-08
123226		Glycoside hydrolase	37	Candidate α,α-trehalase	-1.0944	0.000000518
2735	chi18-6	Glycoside hydrolase	18	Candidate chitinase	-1.0999	0.000000414
49081	cel74a	Glycoside hydrolase	74	Xyloglucanase	-1.3971	6.56E-15
45717	mds1	Glycoside hydrolase	47	α-1,2-mannosidase	-1.5459	2.41E-09
123232	egl3/cel12 a	Glycoside hydrolase	12	Endo-β-1,4-glucanase	-1.6085	3.92E-18
79602		Glycoside hydrolase	81	Candidate endo-1,3-β-glucanase	-1.6627	4.93E-19
44366		Carbohydrate esterase	3	Candidate esterase	-1.8139	3.39E-32
120312	egl2/cel5a	Glycoside hydrolase	5	Endo-β-1,4-glucanase	-1.8718	8.51E-43
123818	xyn2	Glycoside hydrolase	11	Endo-β-1,4-xylanase	-1.9745	3.37E-26
69944		Glycoside hydrolase	31	Candidate α-xylosidase/α-glucosidase	-2.0070	3.94E-17
111849	xyn4	Glycoside hydrolase	30	Endo-β-1,4-xylanase	-2.0563	5.74E-46
74807		Glycoside hydrolase	76	Candidate α-1,6-mannanase	-2.7202	5.29E-89
123283	abf1	Glycoside hydrolase	54	α-L-arabinofuranosidase I	-2.7247	1.3E-48
122495		Glycoside hydrolase	76	Candidate α-1,6-mannanase	-3.0661	1.33E-53
120229	xyn3	Glycoside hydrolase	10	Endo-β-1,4-xylanase	-3.3849	6.11E-45
62166		Glycoside hydrolase	2	Candidate β-mannosidase	-3.4682	5.64E-26
120961	cel61b	Glycoside hydrolase	61	Candidate copper-dependent polysaccharide monooxygenase	-3.5574	1.35E-85
76210	abf2	Glycoside hydrolase	62	Candidate α-L-arabinofuranosidase	-3.8807	1.08E-65
56996	man1	Glycoside hydrolase	5	β-Mannanase	-5.4028	5.28E-84
124175		Glycoside hydrolase	64	Candidate endo-1,3-β-glucanase	-5.6390	2.01E-137
55886		Glycoside hydrolase	16	Candidate glucan endo-1,3(4)-β-D-glucosidase	-7.0670	3.59E-67

Table S4C. CAZy enzymes that are up regulated in glucose

Protein ID	Name	Class	Family	Annotation	log_2 FC gluc/soph/cel	p-value
121136		Glycoside hydrolase	NA	-	-5.1769	0.022304559
49274		Glycoside hydrolase	16	Candidate glucan endo-1,3(4)-β-D-glucosidase	-3.6300	0.00598636
121294		Glycoside hydrolase	16	Candidate glucan endo-1,3(4)-β-D-glucosidase	-2.6584	0.005798218
50215		Glycoside hydrolase	16	Candidate endo-1,3-β-D-glucosidase/1,3-glucan binding protein	-2.5328	0.0007701
82616	cel5b	Glycoside hydrolase	5	Candidate membrane bound endoglucanase	-2.4871	0.000368036
82633		Glycoside hydrolase	72	Candidate β-1 3-glucanosyltransferase	-2.1732	1.15E-15
82633		Glycoside hydrolase	72	Candidate β-1 3-glucanosyltransferase	-2.0714	0.002177737
122511		Glycoside hydrolase	16	Candidate glucan endo-1,3(4)-β-D-glucosidase	-2.0364	3.25E-12
53731		Glycoside hydrolase	5	Candidate endo-β-1,4-glucanase	-2.0080	0.0000499
39942		Glycoside hydrolase	17	Candidate glucan endo-1,3-β-glucosidase	-1.9124	0.030050127
66041	chi18-18	Glycoside hydrolase	18	Candidate chitinase	-1.5663	1.06E-19
39755		Glycoside hydrolase	16	Candidate glucan endo-1,3(4)-β-D-glucosidase	-1.3516	0.002774499
77284		Glycoside hydrolase	12	Candidate endo-β-1,4-glucanase	-1.3141	0.000101813
65333		Glycoside hydrolase	15	Candidate alpha-glycosidase (Glucoamylase and related glycosyl hydrolases)	-1.3056	0.000892332
81598	chi18-7	Glycoside hydrolase	18	Candidate chitinase	-1.1877	0.0000114
68064		Glycoside hydrolase	43	Candidate β-xylosidase/α-L-arabinofuranosidase	-1.1055	0.0000069
71399		Glycoside hydrolase	16	Candidate endo-1,3-β-glucanase	-1.0620	0.000340308
