# Supplementary material for: Comparative metabolism of cellulose, sophorose and glucose in Trichoderma reesei using high-throughput genomic and proteomic analyses
Source: Biotechnol Biofuels. 2014 Mar 21;7:41. doi: 10.1186/1754-6834-7-41 (PMC3998047; doi:10.1186/1754-6834-7-41)
Supplement: Additional file 5: Table S5 — The main transcription factors genes induced in presence of cellulose, sophorose and glucose. [file 1754-6834-7-41-S5.pdf]

Table S5. The main transcription factors genes induced in presence of cellulose, sophorose and glucose.

Condition	Protein ID	Description	Cellulose/Glucose	Sophorose/Cellulose	Sophorose/Glucose	Regulation
**Cellulose**	110152	BZIP transcriptional regulator	2.451163917	-0.954817566	ns*	↑
	120698	C2H2 transcriptional regulator	2.123441639	-0.537738419	ns	↑
	108775	Transcription factor AbaA	1.69505647	-1.765426546	ns	↑
	68254	Zn2Cys6 transcriptional regulator	1.334731053	-1.507788361	ns	↑
	69972	Zn2Cys6 transcriptional regulator	1.850729507	-1.073975388	ns	↑
	121164	Zn2Cys6 transcriptional regulator	1.753882783	-1.185998572	ns	↑
	105269	Zn2Cys6 transcriptional regulator	2.836027951	-1.985112694	ns	↑
**Sophorose**	73654	BZIP transcriptional regulator	ns	1.253232624	1.962791449	↑
	67418	C2H2 transcription factor	ns	0.602927396	1.696446819	↑
	120224	C2H2 transcriptional regulator	ns	0.763324449	1.588719202	↑
	120428	C2H2 transcriptional regulator	ns	0.618870167	1.282988789	↑
	120908	myb transcriptional regulator	ns	1.007563834	0.898357676	↑
	80200	transcription factor (Snd1/p100)	ns	1.479160255	0.930539936	↑
	62244	Zn2Cys6 transcriptional regulator	ns	1.898437819	1.90912878	↑
	65746	Zn2Cys6 transcriptional regulator	ns	0.818299772	1.216395359	↑
	66828	Zn2Cys6 transcriptional regulator	ns	1.001891693	0.692445314	↑
	68455	Zn2Cys6 transcriptional regulator	ns	0.976501182	1.468742399	↑
	55274	Zn2Cys6 transcriptional regulator	ns	1.478449882	2.75481939	↑
	70351	Zn2Cys6 transcriptional regulator	ns	0.791626879	2.862164185	↑
	58389	Zn2Cys6 transcriptional regulator	ns	0.956864949	1.693906081	↑
	21997	Zn2Cys6 transcriptional regulator	ns	1.296895562	1.435194535	↑
	123881	Zn2Cys6 transcriptional regulator	ns	2.243009482	3.209907306	↑
	121107	Zn2Cys6 transcriptional regulator	ns	1.680571487	1.881050039	↑
	73792	Zn2Cys6 transcriptional regulator	ns	1.039341237	1.0882947	↑
	72611	Zn2Cys6 transcriptional regulator	ns	0.780621626	2.412921306	↑

**Glucose**	119759	BZIP transcriptional regulator	-1.408395287	ns	-1.599377882	↑
	21270	CAP20 virulence factor	-1.358418134	ns	-1.41606604	↑
	78049	Elongation factor Tu (G)	-1.331430435	ns	-1.341998166	↑
	75472	Transcriptional regulator, unknown	-0.888154734	ns	-1.003172336	↑
	74346	Translation elongation factor precursor from Aspergillus fumigatus	-1.341448718	ns	-1.809810453	↑
	57676	Translation initiation factor 3, subunit i (elf-3i)	-1.162225859	ns	-0.924258348	↑
	74252	Translation initiation factor 6 (eIF6) by homologyToThe corresponding protein in other eukaryotes	-0.760381566	ns	-1.027525189	↑
	54437	Zn2Cys6 transcriptional regulator	-1.279794868	ns	-2.05063782	↑
	66047	Zn2Cys6 transcriptional regulator	-1.210931508	ns	-1.723538694	↑
	55759	Zn2Cys6 transcriptional regulator	-1.027889564	ns	-0.799460218	↑
	57534	Zn2Cys6 transcriptional regulator	-0.872730004	ns	-1.144605108	↑
	102497	Zn2Cys6 transcriptional regulator	-3.879551717	ns	-3.702652263	↑
	102499	Zn2Cys6 transcriptional regulator	-4.3853748	ns	-3.33778682	↑
	109394	Zn2Cys6 transcriptional regulator	-3.224569925	ns	-4.109305834	↑
	104182	Zn2Cys6 transcriptional regulator	-1.672788142	ns	-2.477423167	↑
	112202	Zn2Cys6 transcriptional regulator	-2.366445259	ns	-2.830805748	↑
	112499	Zn2Cys6 transcriptional regulator	-4.398421905	ns	-6.818347365	↑
	105520	Zn2Cys6 transcriptional regulator	-0.885197724	ns	-1.405556118	↑

↑ Up-regulated

*ns Non significant ($p > 0.05$)
