# Supplementary material for: Comparative metabolism of cellulose, sophorose and glucose in Trichoderma reesei using high-throughput genomic and proteomic analyses
Source: Biotechnol Biofuels. 2014 Mar 21;7:41. doi: 10.1186/1754-6834-7-41 (PMC3998047; doi:10.1186/1754-6834-7-41)
Supplement: Additional file 6: Table S6 — Upregulated transporters genes in presence of cellulose, sophorose and glucose. Values are expressed in log2 fold change. [file 1754-6834-7-41-S6.pdf]

Table S6. Up regulated transporters genes in presence of cellulose, sophorose and glucose. Values are expressed in log2 fold change.

Condition	Protein ID	Description	Cellulose/Glucose	Sophorose/Cellulose	Sophorose/Glucose	Regulation
Cellulose	123611	amino acid permease	2.148425243	-1.018627998	*ns	↑
	81442	Amino acid transporters	1.966209469	-1.183045547	ns	↑
	49970	H+ nucleoside cotransporter	1.927586933	-1.259145007	ns	↑
	79644	metal ion transporter SMF2	2.043837749	-1.331583594	ns	↑
	75165	MFS permease	2.296581234	-2.40589709	ns	↑
	69957	MFS permease	11.00123763	-3.672920343	ns	↑
	58561	MFS permease	1.263827312	-1.986055051	ns	↑
	80058	MFS permease	3.709685191	-1.709189491	ns	↑
	74953	MFS permease	2.197302311	-2.734862754	ns	↑
	76800	MFS permease	3.452257121	-2.36050612	ns	↑
	68122	MFS permease	2.598385735	-2.758562575	ns	↑
	79329	MFS permease	1.583101062	-1.069445989	ns	↑
	60116	MRP-type ABC transporter	3.48324532	-1.868888172	ns	↑
	76682	PDR-type ABC transporters	4.171350017	-5.421350611	ns	↑
Sophorose	122124	AAA ATPase	ns	2.799162458	1.832834954	↑
	103149	AAA ATPase	ns	1.794120124	1.942495355	↑
	64710	AAA+-type ATPase	ns	3.313530576	3.041638135	↑
	62693	ABC-transporter Ste6p	ns	1.326318658	2.092948509	↑
	58584	aquaglyceroporin	ns	1.356959982	2.707002687	↑
	81082	aquaglyceroporin	ns	2.339623145	2.672164781	↑
	48444	MFS maltose permease	ns	2.108510434	5.524487374	↑
	69026	MFS permease	ns	1.068202865	4.044022599	↑
	69611	MFS permease	ns	1.51153785	3.123585581	↑
	60945	MFS permease	ns	1.340635975	4.450755008	↑
	77785	MFS permease	ns	1.02121017	1.946694252	↑
	28409	MFS permease	ns	2.547461858	2.988303066	↑

	66111	MRP-type ABC transporter	ns	1.908369236	1.116005778	↑
	80028	MRP-type ABC transporter	ns	1.972475677	3.533756709	↑
Glucose	104106	ADP/ATP carrier protein	-4.320428326	ns	-7.020464427	↑
	67806	Amino acid permease	-1.447097645	ns	-2.047518406	↑
	110316	Amino acid transporters	-5.180375893	ns	-5.473765791	↑
	105752	C4-dicarboxylate transporter/malic acid transport protein	-2.564555841	ns	-4.534235298	↑
	123588	electron transport protein, probably involved in cytochrome C assembly	-1.886537531	ns	-2.868029568	↑
	80879	MFS H+/oligopeptide transporter	-3.907239376	ns	-4.381371479	↑
	43701	MFS multidrug transporter	-4.168612643	ns	-4.77422163	↑
	80086	MFS peptide transporter	-1.343613381	ns	-1.189333931	↑
	66657	MFS permease	-2.3898029	ns	-3.192489619	↑
	69164	MFS permease	-1.191905304	ns	-1.10718336	↑
	59272	MFS permease	-3.192900582	ns	-4.679686587	↑
	60086	MFS permease	-1.705667242	ns	-3.312147864	↑
	21595	MFS permease	-2.557195549	ns	-5.244095186	↑
	78585	MFS permease	-1.062636876	ns	-5.160496132	↑
	76641	MFS permease	-4.454455995	ns	-7.308735362	↑
	76775	MFS permease	-1.411354187	ns	-1.251605107	↑
	107936	MFS permease	-1.817165239	ns	-3.67393342	↑
	108893	MFS permease	-1.063847819	ns	-2.119444967	↑
	26642	MFS permease	-2.621052433	ns	-2.693696614	↑
	78970	mitochondrial (phosphate) carrier	-1.466192984	ns	-2.446292444	↑
	22251	Mitochondrial carnitine-acylcarnitine carrier protein	-1.323889981	ns	-2.237113639	↑
	78679	mitochondrial carrier protein	-1.354266361	ns	-2.299162552	↑
	64818	Mitochondrial substrate carrier	-1.115154896	ns	-1.880375135	↑
	120556	Mitochondrial substrate carrier	-1.353253639	ns	-1.410002871	↑

64920	Monocarboxylate transporter	-3.425556331	ns	-4.854272939	↑
76910	monocarboxylate transporter	-2.151966026	ns	-2.278281217	↑
56176	MRP-type ABC transporter	-1.564655736	ns	-1.546991338	↑
44476	MRP-type ABC transporter	-1.798508093	ns	-1.965381069	↑
59014	PDR-type ABC transporters	-2.192971612	ns	-3.882300536	↑
77703	PDR-type ABC transporters	-3.064829412	ns	-3.787837545	↑

↑ Up-regulated

*ns Non significant ($p > 0.05$)
