# Supplementary material for: Comparative metabolism of cellulose, sophorose and glucose in Trichoderma reesei using high-throughput genomic and proteomic analyses
Source: Biotechnol Biofuels. 2014 Mar 21;7:41. doi: 10.1186/1754-6834-7-41 (PMC3998047; doi:10.1186/1754-6834-7-41)
Supplement: Additional file 7: Table S7 — Differentially expressed genes in cellulose and sophorose. Numbers are expressed as log2 fold change. [file 1754-6834-7-41-S7.pdf]

Table S7. Differentially expressed genes in cellulose and sophorose. Numbers are expressed as log_2 fold change.

Protein ID	Description	Classification	qmSphCel	pqmSphCel
109787	SSCRP	Accessory	-1.4076	1.69E-21
108193	SSCRP	Accessory	-1.1383	0.012829031
50323	OOC1	Accessory	-5.9971	4.76E-56
104584	Epl1/Sm1	Accessory	-1.8653	1.07E-24
21982	Epl1/Sm1	Accessory	1.2598	1.2E-19
106575	GH79 β-glucuronidase	CAZy	1.5174	1.22E-14
58117	GH89 α-N-acetylglucosaminidase	CAZy	1.0084	0.000390284
59082	GH18, chitinaseCHI18-2	CAZy	1.5828	0.003875319
124175	GH64 endo-1,3-β-glucanase	CAZy	-5.6390	2.01E-137
76210	GH62 α-L-arabinofuranosidase ABF2	CAZy	-3.8807	1.08E-65
79602	GH81 endo-1,3-β-glucanase	CAZy	-1.6627	4.93E-19
122495	GH76 α-1,6-mannanase	CAZy	-3.0661	1.33E-53
123283	GH54 α-L-arabinofuranosidase I ABF1	CAZy	-2.7247	1.3E-48
80833	GH18 chitinase Chi18-5	CAZy	-1.0173	3.2E-09
30668	short chain dehydrogenase/reductase	Electron Transport	1.3952	8.99E-15
52337	Thioredoxin-like protein	Electron Transport	-2.4697	0.00000945
69692	Quinoprotein amine dehydrogenase beta chain-like protein	Electron Transport	-1.5444	5.37E-18
78496	Flavin-containing monooxygenase Fmo1 like	Electron Transport	1.2905	5.35E-12
122556	Short-chain dehydrogenase/reductase	Electron Transport	-1.7917	7.19E-24
122948	multicopper oxidase	Electron Transport	-1.1620	5.43E-09
81536	cation transporting ATPase	Electron Transport	-3.6610	2.66E-49
68956	D-aspartate oxidase	Electron Transport	-1.0767	1.03E-12
54166	Cytochrome P450	Electron Transport	-1.0207	0.028063715
67964	Cytochrome P450 CYP2 subfamily	Electron Transport	-1.1030	1.34E-13
57940	alternative oxidase aox1	Electron Transport	-1.8304	0.000146542
81576	Assimilatory sulfite reductase,Alpha subunit	Electron Transport	-1.0943	6.92E-08
68705	cytochrome P450 monooxygenase	Electron Transport	1.8564	4.05E-27

76230	Flavin-containing monooxygenase	Electron Transport	-5.6650	1.42E-08
102487	Cytochrome P450 CYP2 subfamily	Electron Transport	1.2659	0.000000122
54144	ferric reductase	Electron Transport	1.0056	3.33E-12
120357	Zinc-binding oxidoreductase	Electron Transport	-1.0956	3.54E-16
80200	transcription factor (Snd1/p100)	Transcription Factor	1.4792	7.37E-25
76927	translation initiation factor SUI1.	Transcription Factor	-1.0290	6.56E-15
41617	lae1	Transcription Factor	-1.1475	3.76E-10
120908	myb transcriptional regulator	Transcription Factor	1.0076	2.97E-14
122448	C2H2 transcription factor	Transcription Factor	-1.0707	0.00000906
121121	Zn2Cys6 transcriptional regulator	Transcription Factor	1.0117	1.54E-08
106706	Zn2Cys6 transcriptional regulator	Transcription Factor	1.1374	2.22E-10
122271	Zn2Cys6 transcription regulator, C. albicans Fcr1	Transcription Factor	-2.5097	1.66E-49
66828	Zn2Cys6 transcriptional regulator	Transcription Factor	1.0019	4.05E-13
82017	siderophore transporter	Transport	-2.2193	1.18E-58
78465	siderophore transporter	Transport	-1.1121	2.04E-10
46819	MFS hexose transporter	Transport	-1.2472	2.76E-12
65915	MFS permease	Transport	-1.3336	0.0000048
38812	iron transporter	Transport	-1.1778	3.54E-13
3412	Na+/proline symporter PutP	Transport	1.5352	1.35E-27
60988	phosphate transporter	Transport	1.1722	1.37E-11
60987	MRP-type ABC transporter	Transport	1.0530	6.75E-08
79510	MRP-type ABC transporter	Transport	1.0195	1.48E-08
53475	MFS permease	Transport	1.1169	1.72E-10
68869	MFS permease	Transport	-1.5108	1.65E-20
6005	MFS permease	Transport	-1.2256	0.0000512
54865	Amino acid transporters	Transport	-1.6744	1.77E-29
74187	AAA+-type ATPase	Transport	1.5471	1.59E-16
123735	heavy metal translocating P-type ATPase, putative	Transport	-1.7532	1.36E-08
73250	Urea transporter	Transport	-4.0199	9.47E-40

2687	AAA ATPase	Transport	-3.2915	2.33E-08
47987	ZIP Zinc transporter	Transport	-1.1394	0.012513027
108852	unique protein	Unique	1.7271	1.11E-11
108340	unique protein	Unique	1.8002	0.00126811
121396	unique protein	Unique	1.0760	5.28E-09
121336	unique protein	Unique	1.9396	3.9E-30
