# Supplementary material for: Comparative metabolism of cellulose, sophorose and glucose in Trichoderma reesei using high-throughput genomic and proteomic analyses
Source: Biotechnol Biofuels. 2014 Mar 21;7:41. doi: 10.1186/1754-6834-7-41 (PMC3998047; doi:10.1186/1754-6834-7-41)
Supplement: Additional file 8: Table S8 — Comparison of the gene expression levels assayed by RNA-seq and RT-qPCR. The numbers highlighted in red did not correlate. [file 1754-6834-7-41-S8.pdf]

Table S8. Comparison of the gene expression levels assayed by RNA-seq and RT-qPCR. The numbers highlighted in red did not correlated.

Protein ID	Description	RNA-seq log_2 Fold Change			RT-qPCR log_2 Fold Change		
		Cel/Glu	Sph/Cel	Sph/Glu	Cel/Glu	Sph/Cel)	Sph/Glu
58475	unknown protein	-2.81	5.42	2.60	-3.69	4.91	1.22
120975	unknown protein	-3.48	5.32	1.84	-2.88	5.93	3.05
21876	Zinc-binding oxidoreductase	-1.18	5.03	3.83	-1.84	6.24	4.4
104251	unknown protein	-2.70	4.56	1.83	-3.3	5.95	2.65
110267	unknown protein	-3.18	4.49	1.29	-2.39	4.96	2.57
76359	unknown protein, only present in ascomycota	-1.00	4.41	3.40	-3.63	3.92	0.29
122792	unknown protein	-1.72	4.27	2.54	-0.2	5.17	4.97
106314	Ankyrin	-3.01	3.37	0.33	-3.12	4.07	0.95
64710	AAA+-type ATPase	-0.27	3.31	3.04	-1.72	3.24	1.52
109945	unique protein	-1.24	3.11	1.86	-3.5	2.67	-0.83
72379	conidiospore surface protein cmp1	6.12	-6.12	-1.72	7.93	-5.67	2.26
82227	GH3 β-glucosidase CEL3c	3.80	1.69	5.47	4.96	1.98	6.94
123232	GH12 endo-ß-1,4-glucanase	11.42	-1.61	9.79	16.63	-0.88	15.75
120961	GH61 polysaccharide monooxygenase CEL61b	11.42	-3.56	7.84	18.78	-2.42	16.36
72567	GH6 Cellobiohydrolase CEL6A/CBH2	9.66	-0.13	9.50	16.94	-0.02	16.92
123989	GH7 Cellobiohydrolase CBH1/CEL7a	9.44	0.17	9.59	17.42	0.11	17.53
123992	swollenin	5.53	0.59	6.09	6.61	0.14	6.75
73643	GH61 polysaccharide monooxygenase CEL61a	7.73	-0.66	7.05	9.07	-1.12	7.95
22197	GH1 ß-glucosidase CEL1b	5.00	1.16	6.14	6.52	1.34	7.86
121735	GH3 β-glucosidase CEL3b	4.11	1.21	5.31	8.02	0.12	8.14
