# Supplementary material for: Comparative metabolism of cellulose, sophorose and glucose in Trichoderma reesei using high-throughput genomic and proteomic analyses
Source: Biotechnol Biofuels. 2014 Mar 21;7:41. doi: 10.1186/1754-6834-7-41 (PMC3998047; doi:10.1186/1754-6834-7-41)
Supplement: Additional file 11: Table S1 — Primers used in the validation of differentially expressed genes. [file 1754-6834-7-41-S11.pdf]

Table S1. Primers used in the validation of differentially expressed genes.

Protein ID	Sequence 5'→3' (Foward)	Sequence 5'→3' (Reverse)
58475	ACTTCCAAGGTCACAGGTTG	AGTCCGTTCAACATGACCTG
120975	GTCGTTGTGAGAACTGCAATG	ACCCAGAAATGAAAGCCTCC
21876	GTCGGGTCGTTGACTATAACAC	ATTGCTCTTCACCGTCTTCC
104251	CATTTTGGGCTGGGACTCTAG	TTCAAAGTACTGCTCAGGGC
110267	TGGAATACAATCAGGCGGTG	CGGTTGGAATTCTGCTTTGG
76359	GGGCTAGAGAATTGGAAGACG	ATCTCGCCTCCAAATCCAAG
122792	TCTCGTCAACAGGCAATCAG	CAAGCCGTCCAGTTTTACATG
106314	GTGGTCGTGACTGAGATTGTC	AATTGCCTCTCCCTTGGTG
123673	AGTCATTGATGGAGCTTCTGG	CACTTTGCGTTCTTGTTCTGTC
109945	CTGAAGCATGGATACGAGGTC	AGTAGATCCAAACCAAGGCAAG
72379	ACTCTGAATGCAAGTCTGGAC	TGGTCCTTTTCGCAGTATCC
82227	GCGTACAATGGCATCAATGG	ATCCCAACCCCATTCCTTTC
123232	AGATTGCCATTCCCCAGAAG	CCCGAGTACGTGACATGATTC
120961	ACTATGTCTTCCGCCATGAAC	CACGCACTGAGGATAGTTCTG
72567	ACAAGAATGCATCGTCTCCG	TGTTCCACCCGTTGTAGTTG
123989	CCGAGCTTGGTAGTTACTCTG	GGTAGCCTTCTTGACTGAGT
123992	CCAAACTATACGAGTAGCC	GAGTGAATGTCTTGATGG
73643	GCGCCACTGTTCCTGGAG	ACCGCTGCCACCACACTG
22197	CCATCTACATCACCGAGAACG	TCCAAGTGCGAGTCAAAGTAG
121735	CCAGGATAACTTCAACGAGGG	ATGTGGAGGTTGGAGAACTTG
44504	TGGATCGTCAACTGGTTCTACGA	GCATGTGTAGCAACGTGGTCTTT
61470	TGAGAGCGGTGGTATCCACG	GGTACCACCAGACATGACAATGTT
